# Named Entity Recognition and Relation Detection for Biomedical Information Extraction

**DOI:** 10.3389/fcell.2020.00673

**Published:** 2020-08-28

**Authors:** Nadeesha Perera, Matthias Dehmer, Frank Emmert-Streib

**Affiliations:** ^1^Predictive Society and Data Analytics Lab, Faculty of Information Technology and Communication Sciences, Tampere University, Tampere, Finland; ^2^Department of Mechatronics and Biomedical Computer Science, University for Health Sciences, Medical Informatics and Technology (UMIT), Hall in Tirol, Austria; ^3^College of Artificial Intelligence, Nankai University, Tianjin, China; ^4^Faculty of Medicine and Health Technology, Institute of Biosciences and Medical Technology, Tampere University, Tampere, Finland

**Keywords:** natural language processing, named entity recognition, relation detection, information extraction, deep learning, artificial intelligence, text mining, text analytics

## Abstract

The number of scientific publications in the literature is steadily growing, containing our knowledge in the biomedical, health, and clinical sciences. Since there is currently no automatic archiving of the obtained results, much of this information remains buried in textual details not readily available for further usage or analysis. For this reason, natural language processing (NLP) and text mining methods are used for information extraction from such publications. In this paper, we review practices for Named Entity Recognition (NER) and Relation Detection (RD), allowing, e.g., to identify interactions between proteins and drugs or genes and diseases. This information can be integrated into networks to summarize large-scale details on a particular biomedical or clinical problem, which is then amenable for easy data management and further analysis. Furthermore, we survey novel deep learning methods that have recently been introduced for such tasks.

## 1. Introduction

With the exploding volume of data that has become available in the form of unstructured text articles, Biomedical Named Entity Recognition (BioNER) and Biomedical Relation Detection (BioRD) are becoming increasingly important for biomedical research (Leser and Hakenberg, 2005). Currently, there are over 30 million publications in PubMed (Bethesda, 2005) and over 25 million references in Medline (Bethesda, 2019). This amount makes it difficult to keep up with the literature even in more specific specialized fields. For this reason, the usage of BioNER and BioRD for tagging entities and extracting associations is indispensable for biomedical text mining and knowledge extraction.

Named-entity recognition (NER), in general, (also known as entity identification or entity extraction) is a subtask of information extraction (text analytics) that aims at finding and categorizing specific entities in text, e.g., nouns. The phrase “*Named Entity”* was coined in 1996 at the *6th Message Understanding Conference* (MUC) when the extraction of information from unstructured text became an important problem (Nadeau and Sekine, 2007). In the linguistic domain, Named Entity Recognition involves the automatic scanning through unstructured text to locate “*entities,”* for term normalization and classification into categories, e.g., as person names, organizations (such as companies, government organizations, committees.), locations (such as cities, countries, rivers) or date and time expressions (Mansouri et al., 2008). In contrast, in the biomedical domain, entities are grouped into classes such as genes/proteins, drugs, adverse effects, metabolites, diseases, tissues, SNPs, organs, toxins, food, or pathways. Since the identification of named entities is usually followed by their classification into standard or normalized terms, it is also referred to as “*Named Entity Recognition and Classification”* (NERC). Hence, both terms, i.e., NER and NERC, are frequently used interchangeably. One reason why BioNER is challenging is the non-standard usage of abbreviations, synonymous, homonyms, ambiguities, and the frequent use of phrases describing “*entities”* (Leser and Hakenberg, 2005). An example of the latter is the neuropsychological condition *Alice in wonderland syndrome*, which requires the detection of a chain of words. For all these reasons, BioNER has undoubtedly become an invaluable tool in research where one has to scan through millions of unstructured text corpora for finding selective information.

In biomedical context, Named Entities Recognition is often followed Relation Detection (RD) (also known as relation extraction or entity association) (Bach and Badaskar, 2007), i.e., connecting various biomedical entities with each other to find meaningful interactions that can be further explored. Due to a large number of different named entity classes in the biomedical field, there is a combinatorial explosion between those entities. Hence, using biological experiments to determine which of these relationships are the most significant ones would be too costly and time-consuming. However, by parsing millions of biomedical research articles using computational approaches, it is possible to identify millions of such associations for creating networks. For instance, identifying the interactions of proteins allows the construction of protein-protein interaction networks. Similarly, one can locate gene-disease relations allowing to bridge molecular information and phenotype information. As such, relation networks provide the possibility to narrow down previously-unknown and intriguing connections to explore further with the help of previously established associations. Moreover, they also provide a global view on different biological entities and their interactions, such as disease, genes, food, drugs, side effects, pathways, and toxins, opening new routes of research.

Despite the importance of NER and RD being a prerequisite for many text mining-based machine learning tasks, survey articles that provide dedicated discussions of how Named Entity Recognition and Relations Detection work, are scarce. Specifically, most review articles (e.g., Nadeau and Sekine, 2007; Goyal et al., 2018; Song, 2018), focus on general approaches for NER that are not specific to the biomedical field or entity relation detection. In contrast, the articles by Leser and Hakenberg (2005) and Eltyeb and Salim (2014) focus only on biomedical and chemical NER, whereas (Li et al., 2013; Vilar et al., 2017) only focus on RD. To address this shortcoming, in this paper, we review both NER and RD methods, since efficient RD depends heavily on NER. Furthermore, we also cover novel approaches based on deep learning (LeCun et al., 2015), which have only recently been applied in this context.

**Figure 1:**
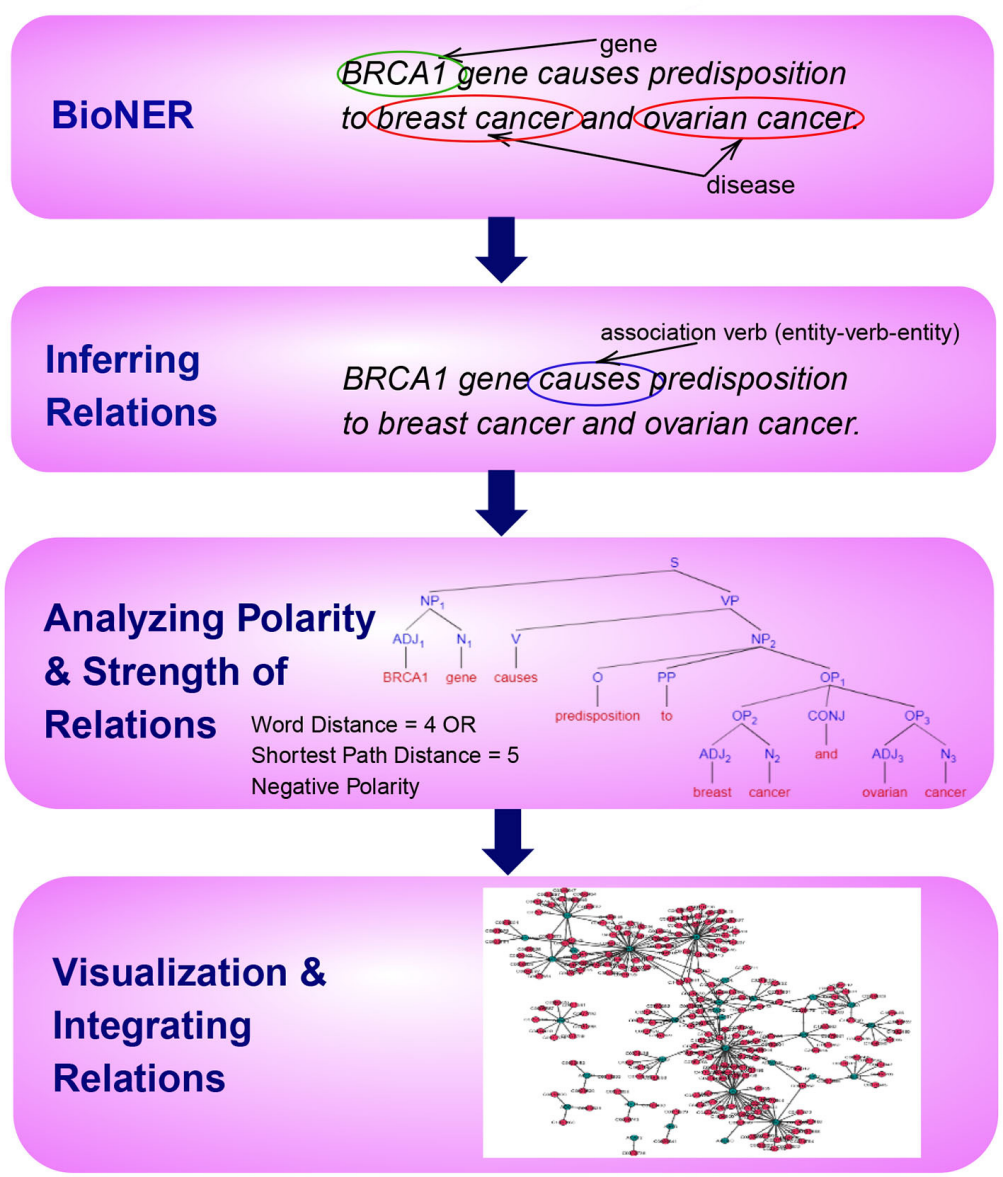
An overview of the principle steps for BioNER and Relation Detection and Analysis. As an example, the sentence “BRCA1 gene causes predisposition to breast cancer and ovarian cancer” is used to visualize each step.

This paper is organized according to the principle steps involved in named entity recognition and relation extraction, shown in Figure 1. Specifically, the first step involves the tagging of entities of biomedical interest, as shown in the figure for the example sentence “*BRCA1 gene causes predisposition to breast cancer and ovarian cancer.”* Here the tagged entities are *BRCA1, Breast Cancer*, and *Ovarian Cancer*. In the next step, relationships between these entities are inferred using several techniques, such as association indicating verbs as illustrated in the example. Here the verb *causes* is identified as pointing to a possible association. In the subsequent step, we aim to distinguish sentence polarity and strength of an inferred relationship. For instance, in the above sentence, the polarity is negative, i.e., indicating an unfavorable relation between the *BRCA1* gene and the tagged disease and the strength of relationship could be extracted by either shortest path in the sentence dependency tree or by a simple word distance as shown in the example. Finally, it is favorable to visualize these extracted relations with their responding strengths in a graph, facilitating the exploration and discovery of both direct associations and indirect interactions, as depicted in Figure 1.

As such, in section 2, we survey biomedical Named Entity Recognition by categorizing different analysis approaches according to the data they require. Then we review relation inferring methods in section 3, strength, and polarity analysis in section 4 and Data Integration and Visualization in section 5. We will also discuss applications, tools, and future outlook in NER and RD in the sections that follow.

## 2. Biomedical Named Entity Recognition (BioNER)

**Figure 2:**
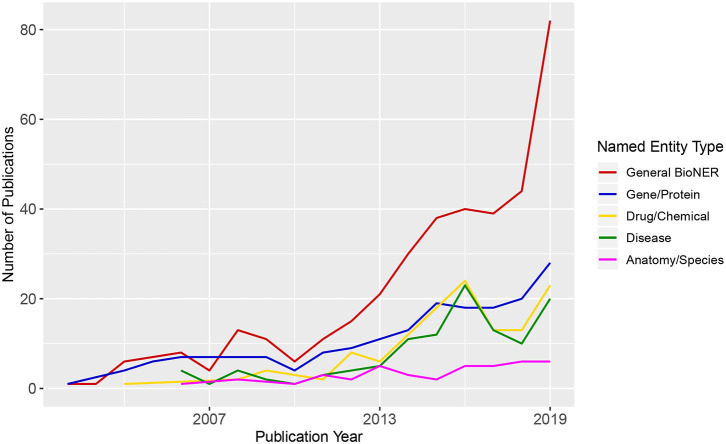
Publication trends in biomedical Named Entity Recognition. The numbers of the published articles were obtained from Web of Science (WoS). The legend shows different queries used for the search of WoS.

BioNER is the first step in relation extraction between biological entities that are of particular interest for medical research (e.g., gene/disease or disease/drug). In Figure 2, we show an overview of trends in BioNER research in the form of scientific publication counts. We extracted the details of the publications that correspond to several combinations of terms related to “*Biomedical Named Entity Recognition”* from the Web of Science (WoS) between 2001 and 2019 and categorize them by general BioNER keywords, i.e., gene/protein, drugs/chemicals, diseases, and anatomy/species. As a result, the counts of articles in each category were plotted chronologically. One can see that there is a steadily increasing amount of publications in general BioNER and a positive growth in nearly every sub-category since the early 2000s. By looking at Figure 2, one can predict that this trend will presumably continue into in the near future.

Accordingly, in the following sections, we discuss challenges in BioNER, the steps in a generic NER pipeline, feature extraction techniques, and modeling methods.

### 2.1. Main Challenges in BioNER

Developing a comprehensive system to capture named entities, requires defining the types on NEs, specific class guidelines for types of NEs, to resolve semantic issues such as metonymy and multi-class entities, and capturing valid boundaries of a NE (Marrero et al., 2013). However, for developing a BioNER system, there are a few more additional problems to overcome than those for general NER (Nayel et al., 2019). Most of these issues are domain-specific syntactic and semantic challenges, hence extending to feature extraction as well as system evaluation. In this section, we will address some of these problems.

Text preprocessing and feature extraction for BioNER requires the isolation of entities. However, as for any natural language, many articles contain ambiguities stemming from the equivocal use of synonyms, homonyms, multi-word/nested NEs, and other ambiguities in naming in biomedical domain (Nayel et al., 2019). For instance, the same entity names can be written differently in different articles, e.g., “*Lymphocytic Leukemia”* and “*Lymphoblastic Leukemia”* (synonyms/British and American spelling differences). Some names may share the same head noun in an article such as in “*91 and 84 kDa proteins”* (nested) corresponding to “*91 kDa protein” and “84 kDa protein”*, in which case the categorization needs to take the context into account. There are various ways for resolving these ambiguities, using different techniques, e.g., name normalization and noun head resolving (D'Souza and Ng, 2012; Li et al., 2017b).

In addition, there are two distinct semantic-related issues resulted from homonyms, metonymy, polysemy, and abbreviations usage. While most terms in the biomedical field have a specific meaning, there are still terms, e.g., for genes and proteins that can be used interchangeably, such as *GLP1R* that may refer to either the gene or protein. Such complications may need ontologies and UMLA concepts to help resolve the class of the entity (Jovanović and Bagheri, 2017). There are also those terms that have been used to describe a disease in layman's terms or drugs that have ambiguous brand names. For example, diseases like *Alice in Wonderland syndrome, Laughing Death, Foreign Accent Syndrome* and drug names such as *Sonata, Yasmin, Lithium* are easy culprits in confusing a bioNER system if there is no semantic analysis involved. For this reason, recent research work (e.g., Duque et al., 2018; Wang et al., 2018d; Pesaranghader et al., 2019; Zhang et al., 2019a) discussed techniques for word sense disambiguation in biomedical text mining.

Another critical issue is the excessive usage of abbreviations with ambiguous meanings, such as “*CLD”*, which could either refer to “*Cholesterol-lowering Drug,” “Chronic Liver Disease,” “Congenital Lung Disease,” or “Chronic Lung Disease.”* Given the differences in the meaning and BioNE class, it is crucial to identify the correct one. Despite being a subtask of word sense disambiguation, authors like (Schwartz and Hearst, 2002; Gaudan et al., 2005) have focused explicitly on abbreviation resolving due to its importance.

Whereas most of the above issues are a result of the lack of standard nomenclature in some biomedical domains, even the most standardized biological entity names can contain long chains of words, numbers and control characters (for example “*2,4,4,6-Tetramethylcyclohexa-2,5-dien-1-one,” “epidemic transient diaphragmatic spasm”*). Such long named-entities make the BioNER task complex, causing issues in defining boundaries for sequences of words referring to a biological entity. However, correct boundary definitions are essential in evaluation and training systems, especially in those where penalizing is required for missing to capture the complete entity (long NE capture) (Campos et al., 2012). One of the most commonly used solutions for multi-word capturing challenge is to use a multi-segment representation (SR) model to tag words in a text as combination of **I**nside, **O**utside, **B**eginning, **E**nding, **S**ingle, **R**ear or **F**ront, using standards like IOB, IOBES, IOE, IOE, or FROBES (Keretna et al., 2015; Nayel et al., 2019).

In order to assess and compare NER systems using gold-standard corpora, it is required to use standardized evaluation scores. A frequently used error measures for evaluating NER is the *F-Score*, which is a combination of Precision and Recall (Mansouri et al., 2008; Emmert-Streib et al., 2019).

Precision, recall, and F-Score are defined as follows (Campos et al., 2012):

(1) $$\begin{array}{r} \text{Precision}=\frac{\text{Relevant Names Recognized}}{\text{Total Names Recognized}}\\ \;=\frac{\text{True Positives}}{\text{True Positives}+\text{False Positives}} \end{array}$$

(2) $$\begin{array}{r} \text{Recall}=\frac{\text{Relevant Names Recognized}}{\text{Relevant Names in Corpus}}\\ \;=\frac{\text{True Positives}}{\text{True Positives}+\text{False Negatives}} \end{array}$$

(3) $$\begin{array}{r} \text{F-score}=2\times \frac{\text{Precision}\times \text{Recall}}{\text{Precision}+\text{Recall}}. \end{array}$$

A problem with scoring a NER system in this way is it requires to define the degree of correctness of the tagged entities for calculating precision and recall. The degree of correctness, in turn, depends on the pre-defined boundaries of the captured phrases. To illustrate this, consider the following example phrase “*Acute Lymphocytic leukemia.”* If the system tags “*lymphocytic leukemia”*, but misses “*Acute”*, we need to decide if it is still a “true positive,” or not. The decision depends on the accuracy requirement of the BioNER; for a system that collects information on patients with Leukemia in general, it may be possible to accept the above tag as a “true positive.” In contrast, if we are looking for rapid progressing Leukemia types, it may be necessary to capture the whole term, including *acute*. Hence, the above would be considered “false positive.”

**Figure 3:**
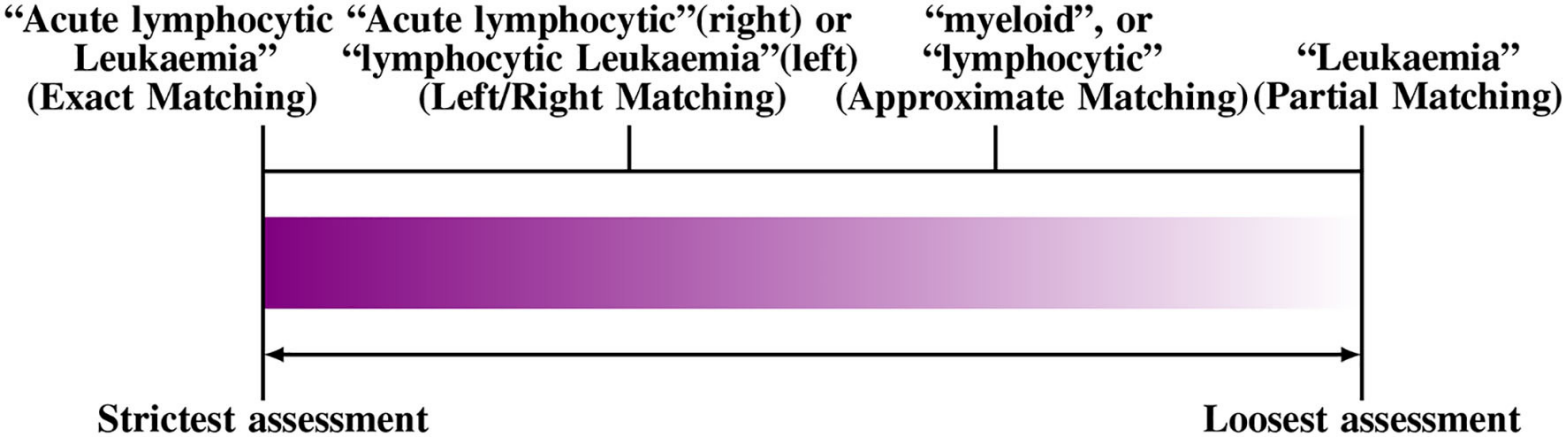
An example for different matching criteria to evaluate Named Entity Recognition. From left to right the criteria become more relaxed (Tsai et al., 2006).

One possible solution is to relax the matching criteria to a certain degree, since an *exact match* criterion tends to reduce the performance of a BioNER system. The effects of such approaches have been evaluated, e.g., using left or right matching, partial or approximate matching, name fragment matching, co-term matching, and multiple-tagging matching. Furthermore, some approaches apply semantic relaxation such as “categorical relaxation,” which merges several entity types to reduce the ambiguity, e.g., by joining DNA, RNA, and protein categories or by combining cell lines and type entities into one class. In Figure 3, we show an example of the different ways to evaluate “*Acute Lymphocytic leukemia*.” For a thorough discussion of this topic, the reader is referred to Tsai et al. (2006).

Until recently, there was also an evaluation-related problem stemming from the scarcity of comprehensively labeled data to test the systems (which also affected the training of the machine learning methods). This scarcity was a significant problem for BioNER until the mid-2000s, since human experts annotated most of the gold standard corpora, and thus were of small size and prone to annotator dependency (Leser and Hakenberg, 2005). However, with growing biological databases and as the technologies behind NER evolved, the availability of labeled data for training and testing have increased drastically in recent years. Presently, there is not only a considerable amount of labeled data sets available, but there are also problem-specific text corpora, and entity-specific databases and thesauri accessible to researchers.

The most frequently used general-purpose biomedical corpora for training and testing are GENETAG (Tanabe et al., 2005), and JNLPBA (Huang et al., 2019), various BioCreative corpora, GENIA (Kim et al., 2003) (which also includes several levels of linguistic/semantic features) and CRAFT (Bada et al., 2012). In Table 1, we show an overview of 10 text corpora often used for benchmarking a BioNER system.

**Table 1:** Benchmark Corpora used for analyzing BioNER systems.

**Corpus**	**Year**	**Text type**	**Training data type**	**Data size**
ChEBI (Shardlow et al., 2018)	2018	Abstracts/Full text	Chemical Entities of Biological Interest	Abs-199/FT-100 (15,000 mentions)
CHEMDNER (Krallinger et al., 2015)	2015	Pubmed Abstracts	Chemicals and Drugs	10,000 (84,355 Entity mentions)
NCBI Disease (Dogan et al., 2014)	2014	Pubmed Abstracts	Diseases	793 (6,892 Disease mentions)
CRAFT (Bada et al., 2012)	2012	Full Text	Cell Type, Chemical Entities of Biological Interest, NCBI Taxonomy, protein, Sequence, Gene, DNA, RNA	97 (140,000 Annotations)
AnEM (Ohta et al., 2012)	2012	Abstracts/ Full text	Pathology, Anatomical Structures/Substances	500 (3,000 mentions)
NaCTeM Metabolite and Enzyme (Nobata et al., 2011)	2011	Medline Abstracts	Metabolites and Enzymes	296
LINNAEUS (Gerner et al., 2010)	2010	Full text Documents	Species	100
GENETAG (Tanabe et al., 2005)	2005	Sentences	Gene, Protein	20,000 Sentences
JNLPBA (Huang et al., 2019)	2004	Abstracts	DNA, RNA, Protein, Cell Type, Cell Line	2,000 (+404 testset)
GENIA (Kim et al., 2003)	2003	Pubmed Abstracts	DNA, RNA, Protein, Cells, Tissue, Anatomy, Organisms, Chemicals	2,000

### 2.2. Principle Steps in BioNER

**Figure 4:**
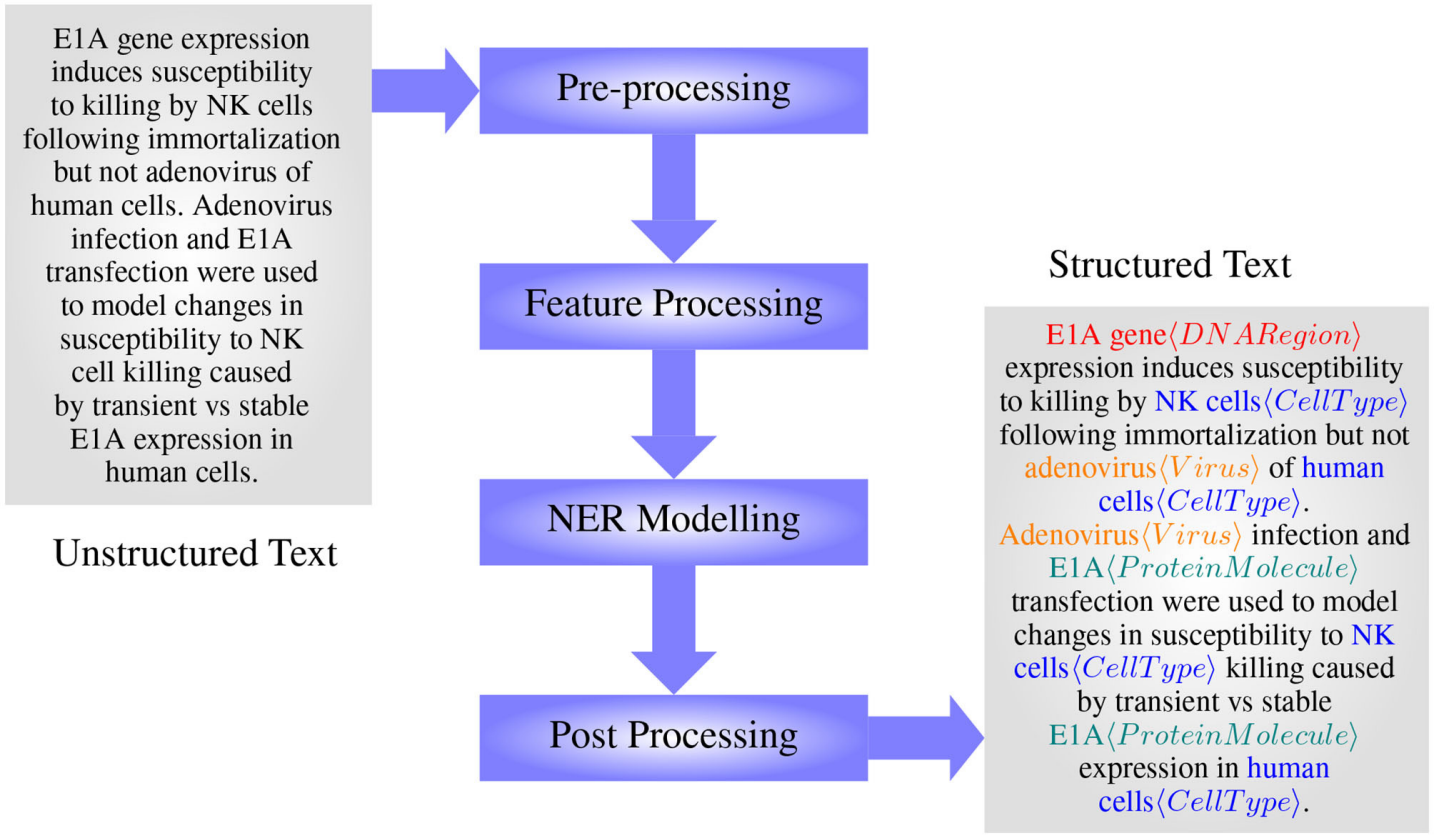
The main steps in designing a BioNER system (with an example from manually annotated GENIA Corpus article MEDLINE:95343554— Routes and Cook, 1995).

The main steps in BioNER include preprocessing, feature processing, model formulating/training, and post-processing, see Figure 4. In the preprocessing stage, data are cleaned, tokenized, and in some cases, normalized to reduce ambiguity at the feature processing step. Feature processing includes different methods that are used to extract features that will represent the classes in question the most, and then convert them into an appropriate representation as necessary to apply for modeling. Importantly, while dictionary and rule-based methods can take features in their textual format, machine learning methods require the tokens to be represented as real-valued numbers. Selected features are then used to train or develop models capable of capturing entities, which then may go through a post-processing step to increase the accuracy further.

#### 2.2.1. Pre-processing

While for general NLP tasks, preprocessing includes steps such as data cleaning, tokenization, stopping, stemming or lemmatization, sentence boundary detection, spelling, and case normalization (Miner et al., 2012), based on the application, the usage of these steps can vary. Preprocessing in BioNER, however, comprises of data cleaning, tokenization, name normalization, abbreviation, and head noun resolving measures to lessen complications in the features processing step. Some studies follow the TTL model (Tokenization, Tagging, and Lemmatization) suggested by Ion (2007) as a standard preprocessing framework for biomedical text mining applications (Mitrofan and Ion, 2017). In this approach, the main steps include sentence splitting and segmenting words into meaningful chunks (tokens), i.e., tokenization, part-of-speech (POS) tagging, and grouping tokens based on similar meanings, i.e., lemmatization using linguistic rules.

#### 2.2.2. Feature Processing

In systems that use rules and dictionaries, orthographic and morphological feature extraction focusing on word formations are the principle choice. Hence, they heavily depend on techniques based on word formation and language syntax. Examples of such include, regular expressions to identify the presence of words beginning with capital letters and entity-type specific characters, suffixes, and prefixes, counting the number of characters, and part-of-speech (POS) analysis to extract nouns/noun-phrases (Campos et al., 2012).

For using machine learning approaches, feature processing is mostly concerned with real-valued word representations (WR) since most machine learning methods require a real-valued input (Levy and Goldberg, 2014). While the simplest of these use bag-of-words or POS tags with term frequencies or a binary representation (one-hot encoding), the more advanced formulations also perform a dimensional reduction, e.g., using clustering-based or distributional representations (Turian et al., 2010).

However, the current state-of-the-art method for feature extraction in biomedical text mining is word embedding due to their sensitivity to even hidden semantic/syntactic details (Pennington et al., 2014). For word embedding, a real-valued vector representing a word is learned in an unsupervised or semi-supervised way from a text corpus. While the groundwork for word embedding was laid by Collobert and Weston (2008), Collobert et al. (2011), over the last few years, much progress has been made in neural network based text embedding taking into account the context, semantics and syntax for NLP applications (Wang et al., 2020). Below we discuss some of the most significant approaches for word representation and word embedding applicable to biomedical field.

##### 2.2.2.1. Rich text features

The most commonly used rich text features in BioNER are Linguistic, Orthographic, Morphological, Contextual, and Lexicon (Campos et al., 2012), all of which are used extensively, when it comes to rule-based and dictionary-based NER modeling. Still, word representation methods may use selected rich text features like char n-grams and contextual information to improve the representation of feature space as well. For instance, char n-grams are used for training vector spaces to recognize rare words effectively in fastText (Joulin et al., 2016), and CBOW in word2vec model uses windowing to capture local features, i.e., the context of a selected token.

To further elaborate, *linguistic features*, generally focus on the grammatical syntax of a given text, by extracting information such as sentence structures or POS tagging. This allows us to obtain tags that are most probable to be a NE since most named entities occur as noun phrases in a text. The *orthographic features*, however, emphasize the word-formation, and as such, attempt to capture indicative characteristics of named entities. For example, the presence of uppercase letters, specific symbols, or the number of occurrences of a particular digit might suggest the presence of a named entity and, therefore, can be considered a feature-token. Comparatively, *morphological features* prioritize the common characteristics that can quickly identify a named entity, for instance, a suffix or prefix. It also uses char n-grams to predict subsequent characters, and regular expression to capture the essence of an entity. *Contextual features* use preceding and succeeding token characteristics of a word by windowing to enhance the representation of the word in question. Finally, *Lexicon features* provides additional domain specificity to named entities. For example, systems that maintain extensive dictionaries with tokens, synonyms, and trigger words that belong to each field are considered to use lexicon features in their feature extraction (Campos et al., 2012).

##### 2.2.2.2. Vector representations of text

**One-hot vector word representation:** The one-hot-encoded vector is the most basic word embedding method. For a vocabulary of size *N*, each word is assigned a binary vector of length *N*, whereas all components are zero except one corresponding to the index of the word (Braud and Denis, 2015). Usually, this index is obtained from a ranking of all words, whereas the rank corresponds to the index. The biggest issue of this representation is the size of the word vector; since for a larger corpus, word vectors are very high-dimensional and very sparse. Besides, frequency and contextual information of each word are lost in this representation but can be vital in specific applications.

**Cluster-based word representation:** In clustering-based word representation, the basic idea is that each cluster of words should contain words with contextually similar information. An algorithm that is most frequently used for this approach is Brown clustering (Brown et al., 1992). Specifically, Brown clustering is a hierarchical agglomerative clustering which represents contextual relationships of words by a binary tree. Importantly, the structure of the binary tree is learned from word probabilities, and the clusters of words are obtained by maximizing their mutual information. The leaves of the binary tree represent the words, and paths from the root to each leaf can be used to encode each word as a binary vector. Furthermore, similar paths and similar parents/grandparents among words indicate a close semantic/syntactic relationship among words. This approach, while similar to a one-hot vector word representation, reduces the dimension of the representation vector, reduces its sparsity, and includes contextual information (Tang et al., 2014).

**Distributional word representation:** The distributional word representation uses co-occurrence matrices with statistical approximations to extract latent semantic information. The first step involves obtaining a co-occurrence matrix, *F* with dimensions *V* × *C*, whereas *V* is the vocabulary size and *C* the context, and each *F*_*ij*_ gives the frequency of a word *i* ∈ *V* co-occurring with context *j* ∈ *C*. Hence, in this approach, it is necessary for the preprocessing to perform stop-word filtering since high frequencies of unrelated words can affect the results negatively. In the second step, a statistical approximation or unsupervised learning function *g*() is applied to the matrix *F* to reduce its dimensionality such that *f* = *g*(*F*), where the resulting *f* is a matrix of dimensions *V* × *d* with *d* ≪ *C*. The rows of this matrix represent the words in the vocabulary, and the columns give the counts of each word vector (Turian et al., 2010).

Some of the most common methods used include clustering (Turian et al., 2010), self-organizing semantic maps (Turian et al., 2010), Latent Dirichlet Allocation (LDA) (Turian et al., 2010), Latent Semantic Analysis (LSA) (Sahlgren, 2006), Random Indexing (Sahlgren, 2006), Hyperspace Analog to Language (HAL) (Sahlgren, 2006). The main disadvantage of these models is that they become computationally expensive for large data sets.

##### 2.2.2.3. Neural network-based text embedding methods

**Figure 5:**
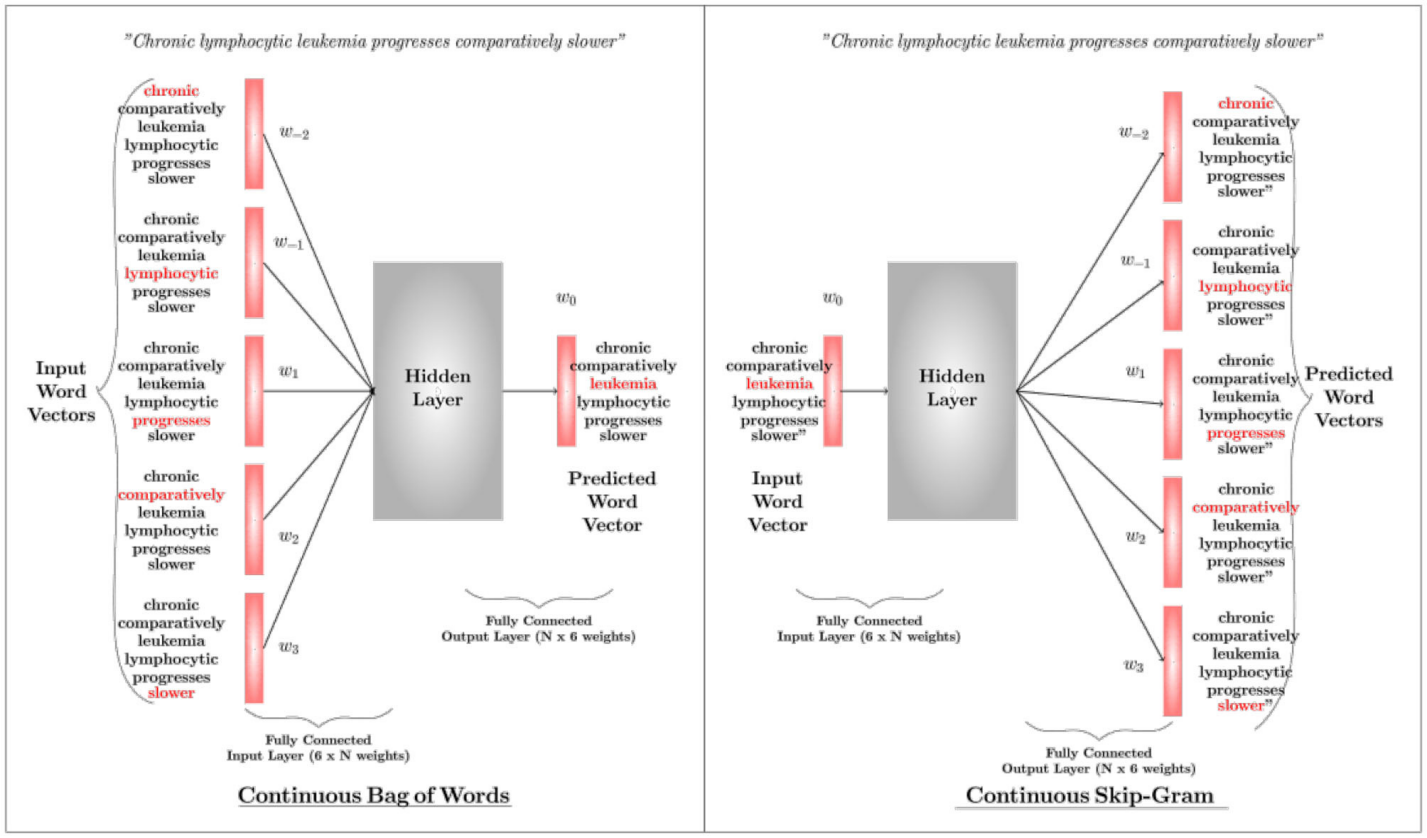
An overview of the Continuous Bag-of-Words Algorithm and the Skip-Gram Algorithm. A CBOW predicts the current word based on surrounding words, whereas Skip-Gram predicts surrounding words based on the current word. Here *w*(*t*) represents a word sequence.

**Word2Vec:** Word2Vec is the state-of-the-art word representation model using a two-layer shallow neural network. It takes a textual corpus as the input, creates a vocabulary out of it, and produces a multidimensional vector representation for each word as output. The word vectors position themselves in the vector space, such that words with a common contextual meaning are closer to each other. There are two algorithms in the Word2Vec architecture, i.e., Continuous Bag-of-Words (CBoW) and Continuous Skip-Gram. Either can be used based on the application requirement. While the former predicts the current word by windowing its close contextual words in the space (with no consideration to the order of those words), the latter uses the current word to predict the words that surround it. The network ultimately outputs either a vector that represents a word (in CBoW) or a vector that represents a set of words (in skip-gram). Figure 5 illustrates the basic mechanisms of the two architectures of word2vec; CBOW and Skip-Gram. Details about these algorithms can be found in Mikolov et al. (2013a,b) (parameter learning of the Word2Vec is explained in Rong, 2014).

**GloVe:** GloVe (Global Vectors) is another word representation method. Its name emphasizes that global corpus-wide statistics are captured by the method, as opposed to word2vec, where local statistics of words are assessed (Pennington et al., 2014).

GloVe uses an unsupervised learning algorithm to derive vector representations for words. The contextual distance among words creates a linear sub-structural pattern in the vector space, as defined by logarithmic probability. The method bases itself on how word-word co-occurrence probabilities evaluated on a given corpus, can interpret the semantic dependence between the words. As such, training uses log-bi-linear modeling with a weighted least-square error objective, where GloVe learns word vectors so that the logarithmic probability of word-word co-occurrence equals the dot product of the words. For example, if we consider two words *i* and *j*, a simplified version of an equation for GloVe is given by

(4) $$\begin{array}{r} w_{i}^{T}\cdot {\tilde{w}}_{j}=log\left(P_{ij}\right)=\frac{X_{ij}}{Xi}. \end{array}$$

Here $w_{i}\in {\mathbb{R}}^{d}$ is the word vector for word *i*, ${\tilde{w}}_{j}\in {\mathbb{R}}^{d}$ is the contextual word vector, which we use to build the word-word co-occurrence. $P_{ij}=P\left(j|i\right)=\frac{X_{ij}}{Xi}$ is the probability of co-occurrence between the words *i* and *j* and *X*_*ij*_ and *X*_*i*_ are the counts of occurrence of word *i* with *j* and occurrence of word *i* alone in the corpus. An in-depth description of GloVe can be found in Pennington et al. (2014).

**fastText:** fastText, introduced by researchers at Facebook, is an extension of Word2Vec. Instead of directly learning the vector representation of a word, it first learns the word as a representation of N-gram characters. For example, if we are embedding the word *collagen* using a 3-gram character representation, the representation would be < *co, col, oll, lla, lag, age, gen, en*>, whereas < and >, indicate the boundaries of the word. These n-grams are then used to train a model to learn word-embedding using the skip-gram method with a sliding window over the word. FastText is very effective in representing suffixes/prefixes, the meanings of short words, and the embedding of rare words, even when those are not present in a training corpus since the training uses characters rather than words (Joulin et al., 2016). This embedding method has also been applied to the biomedical domain due to its ability to generalize over morphological features of biomedical terminology (Pylieva et al., 2018) and detecting biomedical event triggers using fastText semantic space (Wang et al., 2018b).

**BERT/BioBERT:** Bidirectional Encoder Representations for Transformers (BERT) (Devlin et al., 2018), is a more recent approach of text embedding that has been successfully applied to several biomedical text mining tasks (Peng et al., 2019). BERT uses the transformer learning model to learn contextual token embeddings of a given sentence bidirectionally (from both left and right and averaged over a sentence). This is done by using encoders and decoders of the transformer model in combination with Masked Language Modeling to train the network to predict the original text. In the original work targeted for general purpose NLP, BERT was pre-trained with unlabeled data from standard English corpora, and then fine-tuned with task-specific labeled data.

For domain-specific versions of BioBERT (Peng et al., 2019; Lee et al., 2020), one uses the pre-trained BERT model, and by using its learned weights as initial weights, pre-trains the BERT model again with PubMed abstracts and PubMed Central full-text articles. Thereafter, the models are fine-tuned using benchmark corpora, e.g., mentioned in Tables 1, **3**. The authors of BioBERT states that for the benchmark corpora, the system achieves state-of-the-art (or near) precision, recall, and F1 scores in NER and RE tasks.

We would like to highlight that a key difference between BERT, ELMo, or GPT-2 (Peters et al., 2018; Radford et al., 2019) and word2vec or GloVec is that the latter perform a context-independent word embedding whereas the former ones are context-dependent. The difference is that context-independent methods provide only one word vector in an unconditional way but context-dependent methods result in a context-specific word embedding providing more than one word vector representation for one word.

#### 2.2.3. BioNER Modeling

Modeling methods in BioNER can be divided into four categories: Rule-based, Dictionary-based, Machine Learning based, and Hybrid models (Eltyeb and Salim, 2014). However, in recent years, the focus shifted to either pure machine learning approaches or hybrid techniques combining rules and dictionaries with machine learning methods.

While supervised learning methods heavily dominate machine learning approaches in the literature, some semi-supervised and even unsupervised learning approaches are also used. Examples of such work will be discussed briefly later in the section below. The earliest approaches for BioNER focused on Support Vector Machines (SVM), Hidden Markov Models (HMM), and Decision Trees. However, currently, most NER research utilizes deep learning with sequential data and Conditional Random Fields (CRF).

##### 2.2.3.1. Rule-based models

Rule-based approaches, unlike decision trees or statistical methods, use handcrafted rules to capture named-entities and classify them based on their orthographic and morphological features. For instance, it is conventional in the English language to start proper names, i.e., named-entities, with a capital letter. Hence entities with features like upper-case letters, symbols, digits, suffixes, prefixes can be captured, for example, using regex expressions. Additionally, part-of-speech taggers can be used to fragment sentences and capture noun phrases. It is common practice, in this case, to include the complete token as an entity, if at least one part of the token identifies as a named-entity.

An example of the earliest rule-based BioNER system is PASTA (Protein Active Site Template Acquisition, Gaizauskas et al., 2003), in which entity tagging was performed by heuristically defining 12 classes of technical terms, including scope guidelines. Each document is first analyzed for sections with technical text, split into tokens, analyzed for semantic and syntactic features, before extracting morphological and lexical features. The system then uses handcrafted rules to tag and classify terms into 12 categories of technical terms. The terms are tagged with respective classes using the SGML (Standard Generalized Markup Language) format. Recently, however, there is not much literature on pure handcrafted rule-based BioNER systems, and instead, papers such as Wei et al. (2012) and Eftimov et al. (2017) present how combining heuristic rules with dictionaries may result in higher state-of-the-art f-scores. The two techniques complement each other by rules compensating for exact dictionary matches, and dictionaries refining results extracted through rules.

The main drawbacks of rule-based systems are the time-consuming processes involved with handcrafting rules to cover all possible patterns of interest and the ineffectiveness of such rules toward unseen terms. However, in an instance where an entity class is well-defined, it is possible to formulate thorough rule-based systems that can achieve both high precision and recall. For example, most species entity tagging systems rely on binomial nomenclature (two-term naming system of species), which provides clearly defined entity boundaries, qualifying as an ideal candidate for a rule-based NER system.

##### 2.2.3.2. Dictionary-based models

Dictionary-based methods use large databases of named-entities and possibly trigger terms of different categories as a reference to locate and tag entities in a given text. While scanning texts for exactly matching terms included in the dictionaries is a straightforward and precise way of named entity recognition, recall of these systems tends to be lower. Such is the result of increasingly expanding biomedical jargon, their synonyms, spelling, and word order differences. Some systems have been using an inexact or fuzzy matching, by automatically generating extended dictionaries to account for spelling variations and partial matches.

One prominent example of a dictionary-based BioNER model is in the association mining tool **Polysearch** (Cheng et al., 2008), where the system keeps several comprehensive dictionary thesauri, to make tagging and normalization of entities rather trivial. Another example is Whatizit (Rebholz-Schuhmann, 2013), a class-specific text annotator tool available online, with separate modules for different NE types. This BioNER is built using controlled vocabularies (CV) extracted from standard online databases. For instance, *WhatizitChemical* uses a CV from ChEBI and OSCAR3, *WhatizitDisease* uses disease terms CV extracted from MedlinePlus, *whatizitDrugs* uses a CV extracted from DrugBank, *WhatizitGO* uses gene ontology terms and *whatizitOrganism* uses a CV extracted from the NCBI taxonomy. The tool also includes options to extract terms using UniProt databases when using a combined pipeline to tag entities. LINNAEUS, Gerner et al. (2010) is also a dictionary-based NER package designed explicitly to recognize and normalize species name entities in text and includes regex heuristics to resolve any ambiguities. The system has a significant recall of 94% at the mention-level and 98% at the document level, despite being dictionary-based.

More latest state-of-the-art tools have shown preference in using dictionary-based hybrid NER as well, attributing to its high accuracy of performance with previously known data. Moreover, since it involves exact/inexact matching, the main requirement for high accuracy is only a thoroughly composed dictionary of all possible related jargon.

##### 2.2.3.3. Machine learning models

Currently, the most frequently used methods for named entity recognition are machine learning approaches. While some studies focus on purely machine learning-based models, others utilize hybrid systems that combine machine learning with rule-based or dictionary-based approaches. Overall these present state-of-the-art methods.

In this section, we discuss three principal machine learning methodologies utilizing supervised, semi-supervised, and unsupervised learning. These also include Deep Neural Networks (DNN) and Conditional Random Fields (CRF), because newer studies focused on using LSTM/Bi-LSTM coupled with Conditional Random Fields (CRF). Furthermore, in section 2.2.3.4, we will discuss hybrid approaches.

**Supervised methods:** The first supervised machine learning methods used were Support Vector Machines (Kazama et al., 2002), Hidden Markov models (Shen et al., 2003), Decision trees, and Naive Bayesian methods (Nobata et al., 1999). However, the milestone publication by Lafferty et al. (2001) about Conditional Random Fields (CRF) taking the probability of contextual dependency of words into account shifted the focus away from independence assumptions made in Bayesian inference and directed graphical models.

CRFs are a special case of conditionally-trained finite-state machines, in which the final result is a statistical-graphical model that performs well with sequential data, therefore making it ideal for language modeling tasks such as NER (Settles, 2004). In Lafferty et al. (2001), the authors stated that given a text sequence *X* = {*x*_1_, *x*_2_, ..., *x*_*n*_} and its corresponding state label *S* = {*s*_1_, *s*_2_, ...., *s*_*n*_}, the conditional probability of state S for given X can be expressed as:

(5) $$\begin{array}{r} P\left(S|X\right)=\frac{1}{Z_{x}}\exp^{\left(\overset{n}{\underset{i=1}{\sum }}\overset{m}{\underset{j=1}{\sum }}\lambda_{j}f_{j}\left(s_{i-1},s_{i},x,i\right)\right)} \end{array}$$

Here, *s*_*i*_ can be an entity class label (*l* ∈ *L*) for each text *x*_*i*_ (such as a gene or protein), *f*_*j*_(*s*_*i*−1_, *s*_*i*_, *x, i*) is the feature function and λ_*j*_ is the weight vector of *f*_*j*_. Ideally, the learned λ_*j*_ for *f*_*j*_ must be positive for features that correlate to a target label, negative for anti-correlation and zero for irrelevant features. Overall, the learning process for a given training set *D* = {〈*x, l*〉_1_, 〈*x, l*〉_2_, ....., 〈*x, l*〉_*n*_} can be expressed as a log likelihood maximization problem given by:

(6) $$\begin{array}{r} LL\left(D\right)=\overset{n}{\underset{i=1}{\sum }}log\left\{P\left(l_{\left(i\right)}|x_{\left(i\right)}\right)\right\}-\overset{m}{\underset{j=1}{\sum }}\frac{\lambda_{j}^{2}}{2\sigma^{2}} \end{array}$$

Modified Viterbi algorithm assigns respective labels for the new data, after the training process (Lafferty et al., 2001).

**Deep learning:** In the last 5 years, there is a shift in the literature toward general deep neural network models (LeCun et al., 2015; Emmert-Streib et al., 2020). For instance, feed-forward neural networks (FFNN) (Furrer et al., 2019), recurrent neural networks (RNN), or convolution neural networks (CNN) (Zhu et al., 2017) have been used for BioNER systems. Among these, frequent variations of RNNs are, e.g., Elman-type, Jordan-type, unidirectional, or bidirectional models (Li et al., 2015c).

The Neural Network (NN) language models are essential since they excel at dimension reduction of word representations and thus help improve performances in NLP applications immensely (Jing et al., 2019). Consequently, Bengio et al. (2003) introduced the earliest NN language model as a feed-forward neural network architecture focusing on “fighting the curse of dimensionality.” This FFNN that first learns a distributed continuous space of word vectors is also the inspiration behind CBOW and Skip-gram models of feature space modeling. The generated distributed word vectors are then fed into a neural network, that estimates the conditional probability of each word occurring in context to the others. However, this model has several drawbacks, first being that it is limited to pre-specifiable contextual information. Secondly, it is not possible to use timing and sequential information in FFNNs, which would facilitate language to be represented in its natural state, as a sequence of words instead of probable word space (Jing et al., 2019).

In contrast, convolutional neural networks (CNN) are used in literature as a way of extracting contextual information from embedded word and character spaces. In Kim et al. (2016), such a CNN has been applied to a general English language model. In this setup, each word is represented as character embeddings and fed into a CNN network. Then the CNN filters the embeddings and creates a feature vector to represent the word. Extending this approach to Biomedical text processing, Zhu et al. (2017), generates embeddings for characters, words, and POS tagging, which are then combined to represent words and fed to a CNN level with several filters. The CNN outputs a vector representing the local feature of each term, which can then be tagged by a CRF layer.

To facilitate language to be represented as a collection of sequential tokens, researchers have later started exploring recurrent neural networks for language modeling. Elman-type and Jordan-type networks are such simple recurrent neural networks, where contextual information is fed into the system as weights either in the hidden layers in the former type or the output layer in the latter-type. The main issue with these simple RNNs is that they face the problem of vanishing gradient, which makes it difficult for the network to retain temporal information long-term, as benefited by in a recurrent language model.

Long Short-Term Memory (LSTM) neural networks compensate for both of the weaknesses mentioned in previous DNN models and hence are most commonly used for language modeling. LSTMs can learn long-term dependencies through a special unit called a *memory cell*, which not only can retain information long time but has gates to control which input, output, and data in the memory to preserve and which to forget. Extensions of this are bi-directional LSTMs, where instead of only learning based on past data, as in unidirectional LSTM, learning is based on past and future information, allowing more freedom to build a contextual language model (Li et al., 2016b).

**Figure 6:**
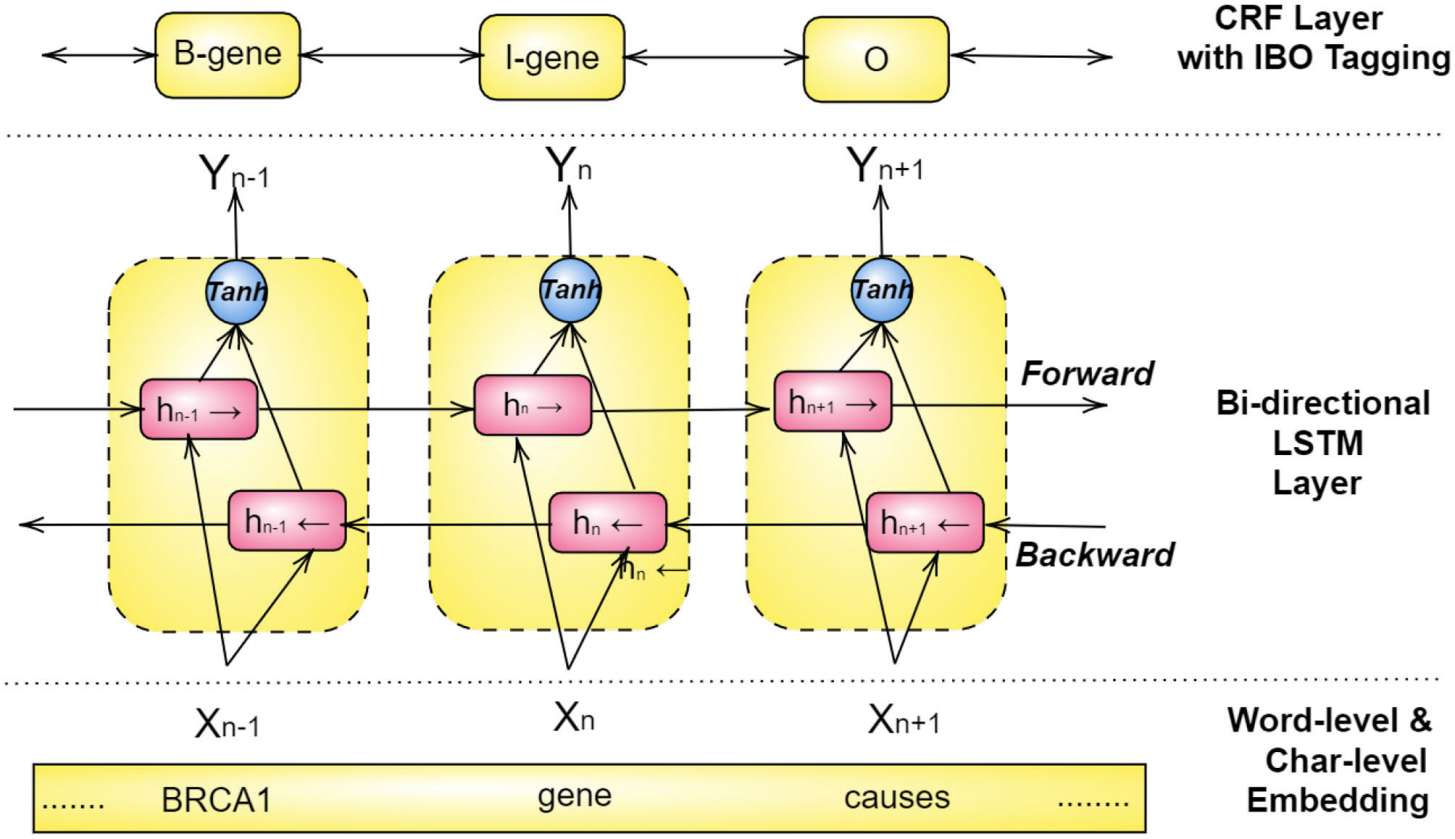
Structure of the Bi-LSTM-CRF architecture for Named Entity Recognition.

For achieving the best results, Bi-LSTM and CRFs models are combined with a word-level and character-level embedding in a structure, as illustrated in Figure 6 (Habibi et al., 2017; Wang et al., 2018a; Giorgi and Bader, 2019; Ling et al., 2019; Weber et al., 2019; Yoon et al., 2019). Here a pre-trained look-up table produces word embeddings, and a separate Bi-LSTM for each word sequence renders a character-level embedding, both of which are then combined to acquire *x*_1_, *x*_2_, ...., *x*_*n*_ as word representation (Habibi et al., 2017). These vectors then become the input to a bi-directional LSTM, and the output of both forward and backward paths, *h*_*b*_, *h*_*f*_, are then combined through an activation function and inserted into a CRF layer. This layer is ordinarily configured to predict the class of each word using an IBO-format (Inside-Beginning-Outside).

If we consider the hidden layer *h*_*n*_ in Figure 6, first, the embedding layer embeds the word *gene* into a vector *X*_*n*_. Next, this vector is simultaneously used as input for the forward LSTM $\vec{h_{n}}$ and the backward LSTM $\overset{⃖}{h_{n}}$, of which the former depends on the past value *h*_*n*−1_ and the latter on the future value *h*_*n*+1_. The combined output resulting from the backward and the forward LSTMs is then passed through an activation function (*tanh*) that results in the output *Y*_*n*_. The CRF layer on the top uses *Y*_*n*_ and tags it as either I-inside, B-Beginning, or O-Outside of a NE (named entity). Consequently, in this example, *Y*_*n*_ is tagged as *I-gene*, i.e., a word inside of the named entity of a gene.

**Semi-supervised methods:** Semi-supervised learning is usually used when a small amount of labeled data and a larger amount of unlabeled data are available, which is often the case when it comes to Biomedical collections. If labeled data is expressed as *X*(*x*_1_, *x*_2_, ...., *x*_*n*_)−>*L*(*l*_1_, *l*_2_, ..., *l*_*n*_) where X is the set of data and L is the set of labels, the task is to develop a model that accurately maps *Y*(*y*_1_, *y*_2_, ..., *y*_*m*_)−>*L*(*l*_1_, *l*_2_, ..., *l*_*m*_) where *m*>*n* and Y is the set of unlabeled data that needs mapping to labels.

Whereas literature using a semi-supervised approach is lesser in BioNER, Munkhdalai et al. (2015) describes how domain knowledge has been incorporated into chemical and biomedical NER using semi-supervised learning by extending the existing BioNER system BANNER. The pipeline runs the labeled and unlabeled data in two parallel lines wherein one line labeled data is processed through NLP techniques to extract rich features such as word and character n-grams, lemma, and orthographic information as in BANNER. In the second line, the unlabeled data corpus is cleaned, tokenized, and run through brown hierarchical clustering and word2vec algorithms to extract word representation vectors, and clustered using k-means. All of the extracted features from labeled and unlabeled data are then used to train a BioNER model using conditional random fields. The authors of this system emphasize that the system does not use lexical features or dictionaries. Interestingly, BANNER-CHEMDNER has shown an 85.68% and an 86.47% F-score on the testing sets of CHEMDNER Chemical Entity Mention (CEM) and Chemical Document Indexing (CDI) sub-tasks and shown a remarkable 87.04% F-score in the test set of the BioCreative II gene-mention task.

**Unsupervised methods:** While unsupervised machine learning has potent in organizing new high throughput data without previous processing and improving the ability of the existing system to process previously unseen information, it is not very often the first choice for developing BioNER systems. However, Zhang and Elhadad (2013) introduced a system, which uses an unsupervised approach to BioNER with the concepts of *seed knowledge* and *signature similarities* between entities.

First, for the seed concepts, semantic types and semantic groups are collected from UMLS (Unified Medical Language System) for each entity type, e.g., protein, DNA, RNA, Cell type, and cell line, to represent the domain knowledge. Second, the candidate corpora are processed using a noun phrase chunker and an inverse document frequency filter, which formulates word sense disambiguation vectors for a given named entity using a clustering approach. The next step generates the signature vectors for each entity class with the intuition that the same class tends to have contextually similar words. The final step compares the candidate named entity signatures and entity class signatures by calculating similarities. As a result, they found the highest F-score of 67.2 for proteins and the lowest at 19.9 for cell-line. Sabbir et al. (2017) used a similar approach, where they implement a word sense disambiguation with an existing knowledge base of concepts extracted through UMLS to develop an unsupervised BioNER model with over 90% accuracy. These unsupervised methods tend to work well when dealing with ambiguous Biomedical entities.

##### 2.2.3.4. Hybrid models

Currently, there are several state-of-the-art applications of BioNER, that combine the best aspects of all the above three methods. Most of these methods combine machine learning with either dictionaries or sets of rules (heuristic/derived), but other approaches exist which combine dictionaries and rule sets as well. Since machine learning approaches have shown to result in better recall values, whereas both dictionary-based and rule-based approaches tend to have better precision values, the former method shows improved F-scores.

For instance, OrganismTagger (Naderi et al., 2011) uses binomial nomenclature rules of naming species to tag organism names in text and combines this with an SVM to assure that it captures organism names that do not follow the binomial rules. In contrast, SR4GN (Wei et al., 2012), which is also a species tagger, utilizes rules to capture species names and a dictionary lookup to reevaluate the accuracy of the tagged entities.

Furthermore, state of the art tools such as Gimli (Campos et al., 2013), Chemspot (Rocktäschel et al., 2012), and DNorm (Leaman et al., 2013) use Conditional Random fields with a thesaurus of own field-specific taxonomy to improve recall. In contrast, OGER++ (Furrer et al., 2019), which performs multi-class BioNER, utilizes a feed-forward neural network structure followed by a dictionary lookup to improve precision.

On the other hand, some systems have been able to combine statistical machine-learning approaches with rule-based models to achieve higher results, as described in this more recent work (Soomro et al., 2017). This study uses the probability analysis of orthographic, POS, n-gram, affixes, and contextual features with Bayesian, Naive-Bayesian, and partial decision tree models to formulate rules of classification.

#### 2.2.4. Post Processing

While not all systems require or use post-processing, it can improve the quality and accuracy of the output by resolving abbreviation ambiguities, disambiguation of classes and terms, as well as parenthesis mismatching instances (Bhasuran et al., 2016). For example, if a certain BioNE is only tagged in one place of the text, yet the same or a co-referring term exist elsewhere in the text, untagged, then the post-processing would make sure these missed NEs are tagged with their respective class. Also, in the case of a partial entity being tagged in a multi-word BioNE, this step would enable the complete NE to be annotated. In the case where some of the abbreviations are wrongly classified or failed to be tagged, some systems use tools such as the BioC abbreviation resolver (Intxaurrondo et al., 2017) at this step to improve the annotation of abbreviated NEs. Furthermore, failure to tag NE also stems from unbalanced parenthesis in isolated entities, which also can be addressed during pre-processing. Interestingly, Wei et al. (2016) describes using a complete rule-based BioNER model for post-processing in disease mention tagging to improve the F-score.

Another important sub-task that is essential at this point, is to resolve coreferences. This may be also important for extracting stronger associations between entities, discussed in the next section. Coreferences are those terms that refer to a named entity without using its proper name, but by using some form of anaphora, cataphora, split-reference or compound noun-phrase (Sukthanker et al., 2020). For example in the sentence “***BRCA1****and* ***BRCA2*** *are proteins expressed in breast tissue where* ***they****are responsible for either restoring or, if irreparable, destroying damaged DNA,”* the anaphora *they* refers to the proteins *BRCA1* and *BRCA2*, and resolving this helps to associate the proteins with their purpose. When it comes to biomedical coreference resolution, it is important to note that generalized methods may not be very effective, given that there are fewer usages of common personal pronouns. Some approaches that have been used in the biomedical text mining literature are heuristic rule sets, statistical approaches and machine learning-based methods. Most of the earlier systems commonly used mention-pair based binary classification and rule-sets to filter coreferences such that only domain significant ones are tagged Zheng et al. (2011a). While the rule set methods have provided state-of-the-art precision they often do not have a high recall. Hence, a sieve-based architecture Bell et al. (2016) has been introduced, which arranges rules starting from high-precision-low-recall to low-precision-high-recall. Recently, deep learning methods have been used for coreference resolution in general domain successfully without using syntactic parsers, for example in Lee et al. (2017). The same system has been applied to biomedical coreference resolution in Trieu et al. (2018) with some domain-specific feature enhancements. Here, it is worth mentioning that the CRAFT corpus, earlier mentioned in Table 1, has an improved version that can be used for coreference resolution for biomedical texts (Cohen et al., 2017).

In the biomedical literature coreference resolution is sometimes conducted (e.g., Zheng et al., 2011b, 2012; Uzuner et al., 2012), but in general underrepresented. A reason for this could be that biomedical articles are differently written in the sense that, e.g., protagonistic gene or protein names are more clearly used and referred to due to their exposed role. However, if this is indeed the reason or if there is an omission in the biomedical NER pipeline requires further investigations.

## 3. Inferring Relations

After BioNER, the identification of associations between the named entities follows. For establishing such associations, the majority of studies use one of the following techniques (Yang et al., 2011): Co-occurrence based approaches, rule-set based approaches, or machine learning-based approaches.

### 3.1. Co-occurrence Based Approaches

The simplest of these methods, co-occurrence based approaches, consider entities to be associated if they occur together in target sentences. The hypothesis is that the more frequent two entities occur together, the higher the probability that they are associated with each other. In an extension of this approach, a relationship is deemed to exist between two (or more) entities if they share an association with a third entity acting as a reciprocal link (Percha et al., 2012).

### 3.2. Rule-Based Approaches

In a rule-based approach, the relationship extraction depends highly on the syntactic and semantic analysis of sentences. As such, these methods rely on part-of-speech (POS) tagging tools to identify associations, e.g., by scanning for verbs and prepositions that correlate two or more nouns or phrases serving as named entities. For instance, in Fundel et al. (2006), the authors explain how syntactic parse trees can be used to break sentences into the form *NounPhase*_1_−*AssociationVerb*−*NounPhrase*_2_, where the *noun phrases* are biomedical entities associated through an *association verb*, and therefore indicates a relationship. In this approach, many systems additionally incorporate a list of verbs that are considered to show implications between nouns, i.e., for example, verbs such as *elevates, catalyzes, influences, mutates*.

**Figure 7:**
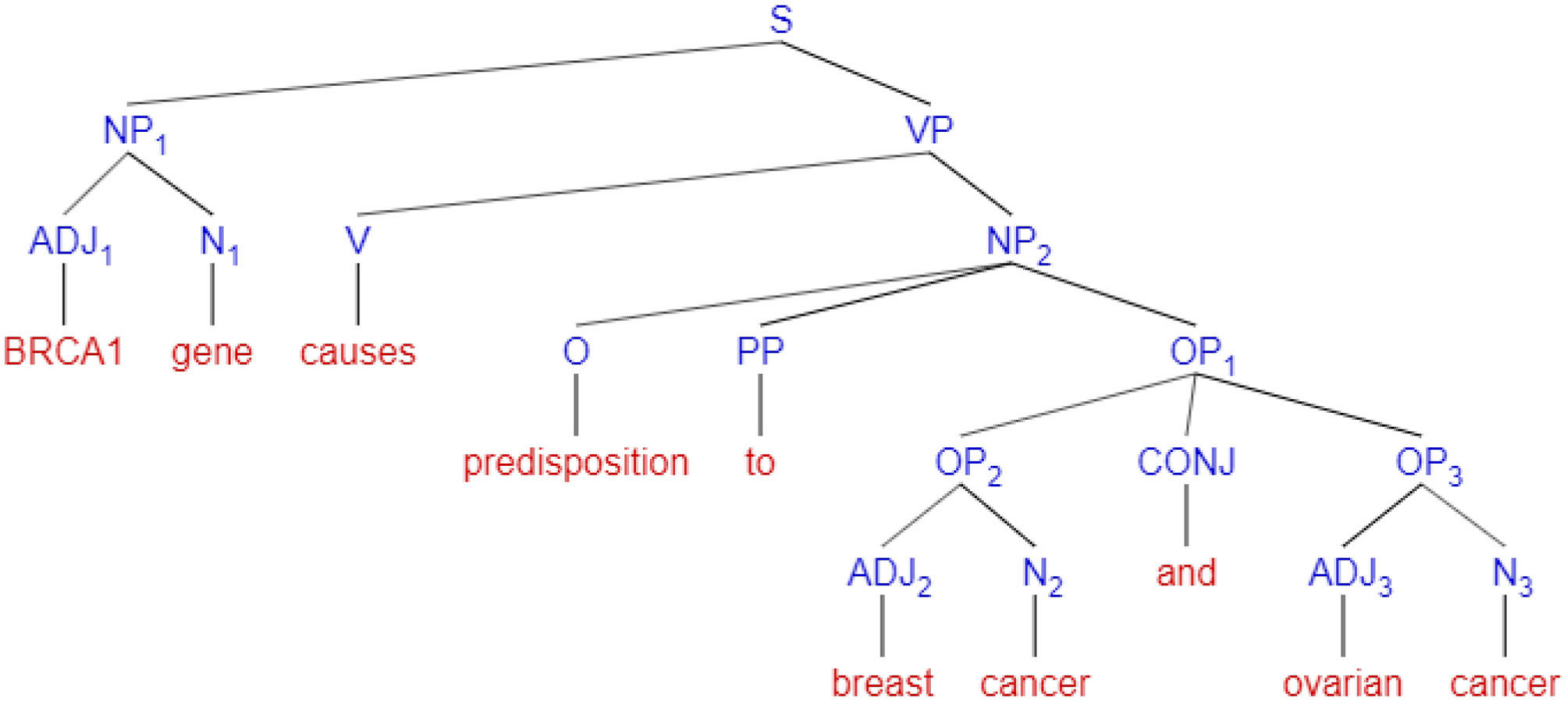
An example of a syntax parse tree for the sentence “*BRCA1 gene causes predisposition to breast cancer and ovarian cancer”*.

In Figure 7, an example of a syntactic sentence parse tree created by POS tagging, is shown. In this figure, nodes signify syntax abbreviations, i.e., S = sentence, NP = Noun Phrase, VP = Verb Phrase, PP = Preposition Phrase, OP = Object of Preposition, CONJ = conjunction ADJ = Adjective, N = Noun, V = Verb, and O = Object. The method first fragments a sentence into noun phrases and verb phrases, and each of these phrases is further segmented to adjectives, nouns, prepositions, and conjunctions for clarity of analysis. More details of the strength of associations will include in section 4.2

### 3.3. Traditional Machine Learning Approaches

The most commonly used machine learning approaches use an annotated corpus with pre-identified relations as training data to learn a model (supervised learning). Previously, the biggest obstacle for using such machine learning approaches for relation detection was acquiring the labeled training and testing data. However, data sets generated through biomedical text mining competitions such as BioCreative and BioNLP have moderated this problem significantly. Specifically, in Table 2, we list a few of the main gold-standard corpora available in the literature for this task.

**Table 2:** Benchmark corpora for biomedical entity relation detection.

**Corpus**	**References**	**Relation type**	**Data content**	**Description**
CHR	Sahu et al., 2019	Chemical-chemical interactions	12,094 PubMed Abstracts and Titles	Chemical Relations database (National Center for Text Mining)
BioInfer	Pyysalo et al., 2007	Gene, RNA, Protein, relations	1100 Sentences	Bio Information Extraction Resource
GE	Kim et al., 2009	Gene and Gene Product Associations	15 Annotated PubMed Articles	Genia Event Extraction Corpus
EU-ADR	Van Mulligen et al., 2012	Diseases, Drugs and Drug Target relations	300 Abstracts 100 for each Entity	European Union - Adverse Drug Reaction Project affiliated
ChEBI	Shardlow et al., 2018	Relations between Chemicals, Proteins, Species, Biological Activity	199 abstracts 100 full papers annotated	Chemical Entities of Biological Interest
BC-II: PPI Corpus	Krallinger et al., 2008	Protein-Protein Interactions	3,536+338 (TR+TE) Related Entries 1,959+339 (TR+TE) Non-Related Entries	BioCreative II - PPI task Corpora
BC-II.5: Elsevier Corpus	Leitner et al., 2010	Protein-Protein Interactions	1190 Articles 124 - PPI positive 1066 - unrelated	BioCreative II.5 Special Corpus provided by Elsevier
BC-V: CDR	Li et al., 2016a	Chemical-Disease relations	1500 PubMed Articles 3116 interactions	BioCreative V - Chemicals and Disease Corpus
BC-VI: ChemProt Corpus	Krallinger et al., 2017	Chemical-Protein interaction	1020+800 (TR+TE) Abstracts	Training/Testing article Corpus for the BioCreative V Task
AIMed Corpus	Bunescu et al., 2005	Protein-Protein interactions	225 Abstracts 200- PPI positive 25- unrelated	Human annotated Corpus for Training to Identify relations

*These data sets contain labeled data that can be used for the training and testing of methods*.

Historically, SVMs have been the first choice for this task due to their excellent performance in text data classification with a low tendency for overfitting. Furthermore, they have also proven to be good with sentence polarity analyzing for extracting positive, negative, and neutral relationships as described by Yang et al. (2011). Of course, in SVM based approaches, feature selection acts as the strength-indicator for accuracy and, therefore, is considered a crucial step in relationship mining using this approach.

One of the earliest studies using an SVM was (Özgür et al., 2008). This study used a combination of methods for evaluating an appropriate kernel function for predicting gene-disease associations. Specifically, the kernel function used a similarity measure incorporating a normalized edit-distances between the paths of two genes, as extracted from a dependency parse tree. In contrast to this, the study by Yang et al. (2011) used a similar SVM model, however, for identifying the polarity of food-disease associations. For this reason, their SVM was trained with positive, negative, neutral, and irrelevant relations, which allowed assigning the polarity in the form of “*risk*.” For instance, particular food can either increase risk, reduce risk, be neutral, or be irrelevant for a disease. Recently, Bhasuran and Natarajan (2018) extended the study by Özgür et al. (2008) using an ensemble of SVMs trained with small samples of stratified and bootstrapped data. This method also included a word2vec representation in combination with rich semantic and syntactic features. As a result, they improved F-scores for identifying disease-gene associations.

Although SVMs appear to take predominance in this task, other machine learning methods have been used as well. For instance, in Jensen et al. (2014), a Naive-Bayes classifier has been used for identifying food-phytochemical and food-disease associations based on TF-IDF (term frequency-inverse document frequency) features. Whereas, in Quan and Ren (2014), a Max-entropy based classifier with Latent Dirichlet Allocation (LDA) was used for inferring gene-disease associations, and in Bundschus et al. (2008) a CRF was used for both NER and relation detection, for identifying disease-treatment and gene-disease associations.

### 3.4. Deep Learning Approaches

Due to the state of the art performance and less need for complicated feature processing, deep learning (DL) methods are becoming increasingly popular for relation extraction in the last five years. The most commonly used DL approaches include convolutional neural networks (CNNs), recurrent neural networks (RNNs), and hybrids of CNN and RNN (Jettakul et al., 2019; Zhang et al., 2019b), most of which are also able to classify relation-type as well.

**Figure 8:**
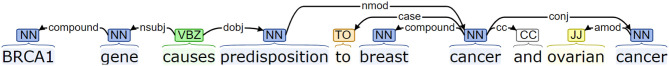
Dependency graph for the sentence “*BRCA1 gene causes predisposition to breast cancer and ovarian cancer”* generated using Standford coreNLP parser (Manning et al., 2014) (nsubj-nominal subject, dobj-direct object, nmod-nominal modifier, amod-adjectival modifier, conj-conjunction, CC-coordinating conjunction, JJ-adjective, NN-noun).

The feature inputs to DL models may include sentence-level, word-level, and lexical-level features represented as vectors (Zeng et al., 2014), positions of the related entities, and the class label of the relation type. The vectors are looked up from pre-trained word and positional vector space on either a single corpus or multiple corpora (Quan et al., 2016). Significantly, the majority of deep learning methods use sentence dependency graphs mentioned in the rule-based approach (Figure 8) to extract the shortest path between entities and relations as features for training (Hua and Quan, 2016a,b; Zhang et al., 2018c; Li et al., 2019). Other studies have used POS tagging, and chunk tagging features in combination with position and dependency paths to improve performance (Peng and Lu, 2017). The models are trained to either distinguish between sentences with relations or to output the type of relation.

The earliest approaches use Convolutional Neural Networks (CNN), where the extracted features e.g., dependency paths/sentences, are represented using the word vector space. Since CNN's require every training example to be of similar size, instances are padded with zeros as required (Liu et al., 2016). After several layers of convolutional operations and pooling, these methods are followed by a fully connected feed-forward neural layer with soft-max activation function (Hua and Quan, 2016b).

Subsequently, LSTM networks, including bi-LSTM, have been used in Sahu and Anand (2018) and Wang et al. (2018e), to learn latent features of sentences. These RNN based models perform well with relating entities that lie far apart from each other in sentences. Whereas, CNNs requires restrictive sized inputs, the RNNs have no such restrains and are useful when long sentences are available, since the input is sequentially processed. These models have been used to extract drug-drug and protein-protein interactions (Hsieh et al., 2017). Extending this further, Zhang et al. (2018c) experiments with bidirectional RNN models using two hierarchical layers, one with two simple RNNs, one with two GRUs, and last with two LSTMs. Here the hierarchical bi-LSTM has shown a better performance.

In recent years, there have also been studies that use a novel approach, i.e., graph convolutional networks (GCN) (Kipf and Welling, 2016) for relation extraction using dependency graphs (Zhang et al., 2018b; Zhao et al., 2019). Graph convolutional networks use the same concept of CNN, but with the advantage of using graphs as inputs and outputs. By using dependency paths to represent text as graphs, GCNs can be applied to relation extraction tasks. In Zhao et al. (2019), the authors use a hybrid model that combines GCNs preceded by bidirectional gated recurrent units (bi-GRU) layer to achieve significant F-measures. Furthermore, for identifying drug-drug interactions, a syntax convolutional neural network has been evaluated for the DDIExtraction 2013 corpus (Herrero-Zazo et al., 2013) and found to outperform other methods (Zhao et al., 2016). Conceptually similar approaches have been used in Suárez-Paniagua et al. (2019); Wei et al. (2019).

In extension, Zheng et al. (2018) uses a hierarchical hybrid model that resembles a reverse CRNN (convolutional recurrent neural network), where a CNN and a soft-max layer follow two bi-LSTM layers. The method has been used to extract chemical-disease relations, and have been trained and evaluated on CDR corpus (Li et al., 2016a). Whereas, authors of Zhang et al. (2018a) uses two CNNs and a bi-LSTM simultaneously to learn from word/relation dependency and sentence sequences, to extract disease-disease and protein-protein relations. These hybrid methods aim to combine the CNN's efficiency in learning local lexical and syntactic features (short sentences) with RNN's ability to learn dependency features over long and complicated sequences of words (long sentences). Both of the above models have been found to perform well with their respective corpora.

### 3.5. Graph-Based Approaches

Graph-based representation preserves the sentence structure by converting the text directly into a graph, where biomedical named entities are vertices and other syntactic/semantic structures connecting them are edges. While complex sentence structures may lead to nested relations, this method facilitates identifying common syntactic patterns indicating significant associations (Luo et al., 2016).

Once the named entities are tagged, the next steps involve splitting sentences, annotating them with POS, and processing other feature extractions as required. Graph extraction is usually performed at this point as a part of the feature extracting process. Once the graphs including concepts and their syntactic/semantic relations are mined, these can be used as kernels, training data for deep learning approaches, or for generating rule sets with the help of graph search algorithms (Kilicoglu and Bergler, 2009; Ravikumar et al., 2012; Panyam et al., 2018a; Björne and Salakoski, 2018). For example, in Liu et al. (2013), approximate subgraph matching has been used to extract biomolecular relations from key contextual dependencies and input sentence graphs. A similar approach has been used in MacKinlay et al. (2013). The paper by Luo et al. (2016) provides a good review including a wide array of examples for which graph-based approaches are used in biomedical text mining.

### 3.6. Hybrid Approaches

Also, the combination of machine learning and graph-based approaches have been studied with great success. For instance, in Kim et al. (2015), a linear graph kernel based on dependency graphs for sentences has been used in combination with an SVM to detect drug-drug interactions. In order to enrich the information captured by kernels, Peng et al. (2015) uses an extended dependency graph that has also been defined to include information beyond syntax. Furthermore, in Panyam et al. (2018b), chemical-induced disease relations have been studied by comparing tree kernels (subset-tree kernel and partial-tree kernel) and graph kernels (all-path-graph and approximate-subgraph-matching). As a result, they found that the all-path-graph kernel performs significantly better in this task.

### 3.7. Others Approaches

In this section, we discuss methods that do not fit in either of the above categories but provide interesting approaches. In Zhou and Fu (2018), an extended variant of the frequency approach is studied, which combines co-occurrence frequency and Inverse Document Frequency (IDF) for relations extraction. The study sets the first precedence to entity co-occurrence in MeSH terms and second to those in the article title, and third to the ones in the article abstract by assigning weights to each precedence level. A vector representation for each document sample is created using these weights for calculating the score of each key-term-association by multiplying IDF with PWK (penalty weight for the keyword, depending on the distance from MeSH root). Next, by comparing with the dictionary entries for relevance, each gene and disease is converted into vectors (*V*_*g*_, *V*_*d*_), and the strength of a relation is calculated through the cosine similarity given by $Cos\langle V_{g},V_{d}\rangle =\frac{V_{g}.V_{d}}{\left|V_{g}|.|V_{d}\right|}$. The authors then evaluate the system by comparing precision, recall, and cosine similarity.

In contrast, the study by Percha and Altman (2015) introduces an entirely novel algorithm to mine relations between entities called Ensemble Clustering for Classification (EBC). This algorithm extract drug-gene associations by combining an unsupervised learning step and a lightly supervised step that uses a small seed data set. In the unsupervised step, all co-occurrences of gene-drugs pairs (*n*) and all dependency path between the pairs (*m*) are mined to create a matrix of *n* × *m* which is then clustered using Information-Theoretic Co-Clustering. The supervised step follows by comparing how often the seed set pairs and test set pairs co-cluster together using a scoring function, and relationships are ranked accordingly. The same authors have extended this method further in Percha and Altman (2018), by applying hierarchical clustering after EBC to extract four types of association between gene-gene, chemical-gene, gene-disease, and chemical-disease. Incidentally, this hierarchical step has enabled additional classification of these relationships into themes such as ten different types of chemical-gene relations or seven distinct types of chemical-disease associations.

## 4. Analyzing Polarity and Strength of Relations

A further refinement following a relation detection is an analysis of the polarity and the strength of the identified associations, providing additional information about the relations and, hence, enhances extracted domain-specific knowledge.

### 4.1. Polarity Analysis

A polarity analysis of relations is similar to a sentiment analysis (Swaminathan et al., 2010; Denecke and Deng, 2015). For inferring the polarity of relations, similar machine learning approaches can be used, as discussed in section 3.3. However, a crucial difference is that for the supervised methods, appropriate training data need to be available, providing information about the different polarity classes. For instance, one could have three polarity classes, namely, positive associations (e.g., *decreases risk, promotes health*), neutral associations (e.g., *does not influence, causes no change*), and negative associations (e.g., *increases risk, mutates cell*). In general, a polarity analysis opens new ways to study research questions of how entities interact with each other in a network. For example, the influence of a given food metabolite on certain diseases can be identified, which may open new courses of food-based treatment regiments (Miao et al., 2012a,b).

### 4.2. Strength Analysis

A strength analysis comes after identifying associations between entities in a text since all extracted events might not be considered significant associations. Especially in simple co-occurrences based method to identify relationships, strength analysis can be vital, since just a simple mention of two entities in a sentence with no explicit reciprocity, may result in them wrongly defined as associations. Some of the most common methods employed in the literature include distance analysis and dependency path analysis, or an extension of those methods.

An example of a method that implements a word distance analysis is Polysearch (Liu et al., 2015). Polysearch is essentially a biomedical web crawler focusing on entity associations. This tool first estimates co-occurrence frequencies and the association verbs to locate content that is predicted to have entity associations. Next, using the word-distances between entity-pairs in the selected text, content relevancy (i.e., the strength of association) score is calculated. Incidentally, this system is currently able to search in several text corpora and databases, using the above method, to find relevant content for over 300 associative combinations of named entity classes.

In Coulet et al. (2010), the authors created syntactic parse trees, as shown in Figure 7, by analyzing sentences selected by the entity co-occurrences approach. Each tree then converts into a directed and labeled dependency graph, whereas nodes are words, and edges are dependency labels. Next, by extracting shortest paths between node pairs in the graph, they transform associations into the form *Verb*(*Entity*_1_, *Entity*_2_), such that *Entity*_1_ and *Entity*_2_ are connected by *Verb*. This approach, which is an extension of the association-identifying method described in section 3.1, hypothesizes that the shortest dependency paths indicate the strongest associations. Other studies that use a dependency analysis of sentences to determine the strength of the associations include (Quan and Ren, 2014; Kuhn et al., 2015; Mallory et al., 2015; Percha and Altman, 2015). Many systems using machine learning approaches, also tend to define syntactic and dependency paths analysis of sentences as a feature selection method before training relation mining models, as discussed in Özgür et al. (2008); Yang et al. (2011), and Bhasuran and Natarajan (2018).

## 5. Visualization and Integrating Relations

### 5.1. Network Visualization

After individual relations between biomedical entities have been inferred, it is convenient to assemble these in the form of networks (Skusa et al., 2005; Li et al., 2014; Kolchinsky et al., 2015). In such networks, nodes (also called vertices) correspond to entities and edges (also called links) to relations between entities. The resulting networks can be either weighted or unweighted. If polarity or strength of relations has been obtained, one can use this information to define the weights of edges as the strength of the relations, leading to weighted networks. Polarity information and relation type classifications can further be used to label edges. For example, these labels could be *positive regulation, negative regulation*, or *transcription*. In this case, edges tend to be directed indicating which entity is influenced by which. Such labeled and/or weighted networks are usually more informative than unweighted ones because they carry more relevant domain-specific information.

The visualization of interaction networks often provides a useful first summary of the results extracted from the relation extraction task. The networks are either built from scratch or automatically by using software tools. Two such commonly used tools for the network visualization are Cytoscape (Franz et al., 2015) and Gephi (Bastian et al., 2009), both providing open-source java libraries. Cytoscape can also be used interactively via a web-interface, while Gephi can be used for 3D rendering of graphs and networks. There are also several libraries specifically developed for network visualization in different languages. For instance, NetbioV (Tripathi et al., 2014) provides an R package and Graph-tool (Peixoto, 2014) a package for Python.

### 5.2. Network Analysis

The networks generated in the above way can be further analyzed to reconfirm known associations, and further explore new ones (Özgür et al., 2008; Quan and Ren, 2014). Measures frequently used for biomedical network analysis include node centrality measures, shortest paths, network clustering, and network density (Sarangdhar et al., 2016). The measures selected to analyze a graph predominantly depend on the task at hand; for example, shortest path analysis is vital for discovering signaling pathways, while clustering analysis helps identify functional subnetwork units. Further commonly used metrics are centrality measures and network density methods, e.g., for identifying the most influential nodes in the network. Whereas graph density compares the number of existing relations between the nodes vs. all possible connections that can be formed in the network, centrality measures are commonly used to identifying the importance of an entity within the entire network (Emmert-Streib and Dehmer, 2011).

There are four main centrality measures, namely, degree, betweenness, closeness, and eigenvector centrality (Emmert-Streib et al., 2018). Degree centrality, the simplest of the above measures, corresponds just to the number of connections of a node. Closeness centrality is given by the reciprocal of the sum of all shortest path lengths between a node and all other nodes in the network, as such it measures the spread of information. Also betweenness centrality utilizes shortest paths by taking into account the information flow of the network. This is realized by counting shortest paths through pairs of nodes. Finally, eigenvector centrality is a measure of influence where each node is assigned a score based on how many other influential nodes are connected to it.

**Figure 9:**
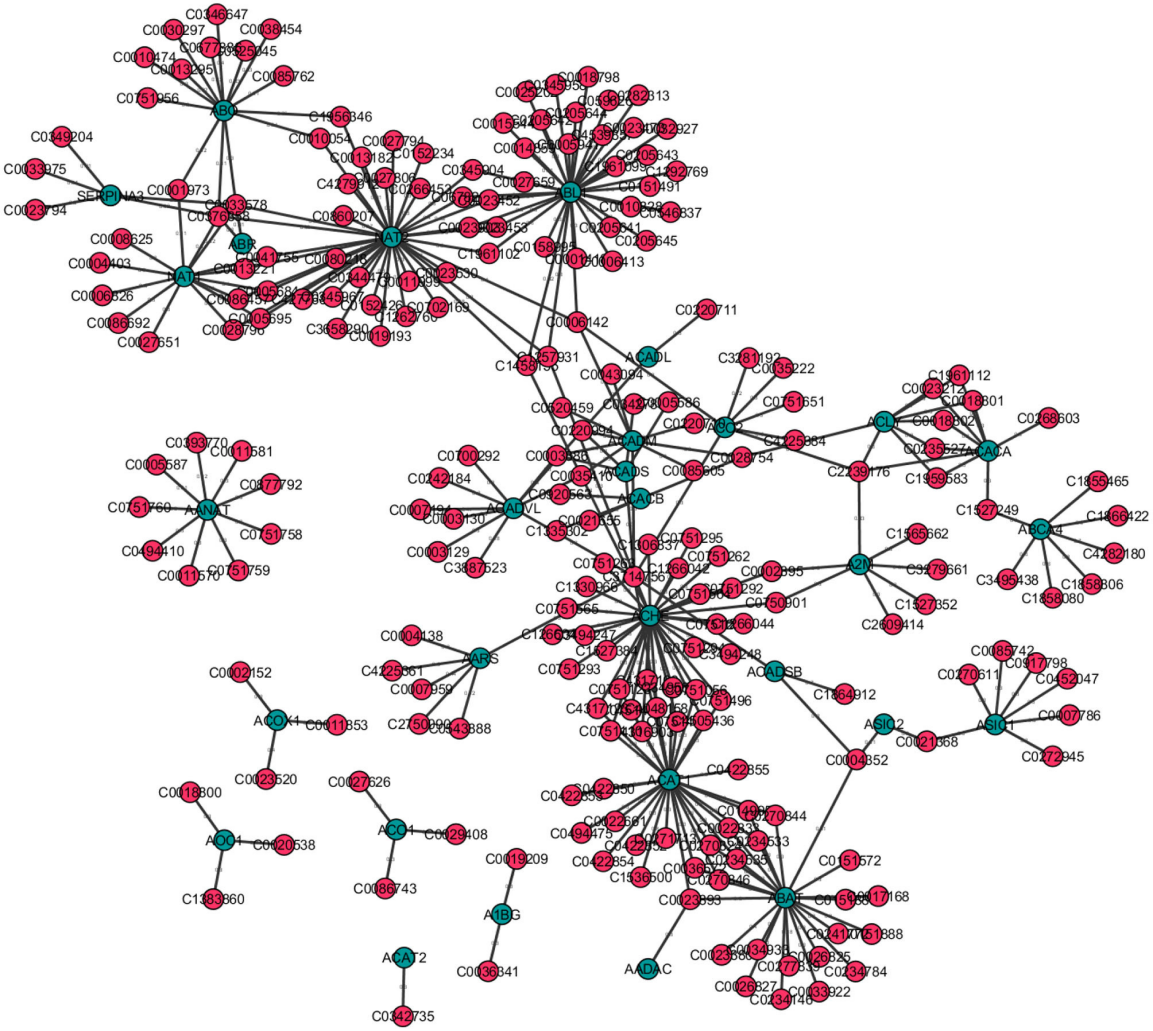
Disease-gene network created for selected 300 entries from the DisGeNet Database (Bauer-Mehren et al., 2010) with Cytoscape v3.7.2. Genes are shown as blue nodes and diseases as pink nodes.

For instance, consider Figure 9, a disease-gene network. Here blue nodes correspond to genes and pink nodes represent diseases. For instance, blue nodes with a higher degree centrality correspond to those genes associated with a higher number of diseases. Similarly, pink nodes with a high degree centrality correspond to diseases that are associated with more genes. Furthermore, the genes with a high closeness centrality are important because they have a direct or indirect association to the largest number of other genes and diseases. Further, if a gene X that is connected to a large number of diseases, and is furthermore connected to gene Y with a high eigenvector centrality, it may be worth exploring if there are diseases in the neighborhood of gene X, that are possibly also associated to gene Y and vice versa. Hence, based on centrality measures, one may be able to find previously undiscovered relations between certain diseases and genes.

## 6. Tools and Data Resources

In this section, we will discuss some of the main benchmark tools and resources available for Named-Entity Recognition and Relation Extraction used in the biomedical domain.

While the training corpora for machine learning methods in BioNER and BioRD both have been discussed extensively in the sections above, here we mention some of the databases with entities and relation mappings. These are crucial for dictionary-based methods and in post-processing, and as such, are often used for biomedical text mining research.

Some of the Named-Entity specific databases that have comprehensive collections of jargon include *Gene Ontology* (Consortium, 2004), *Chemical Entities of Biological Interest* (Shardlow et al., 2018), *DrugBank* (Wishart et al., 2017), *Human Protein Reference Database* (Keshava Prasad et al., 2008), *Online Mendelian Inheritance in Man* (Amberger et al., 2018), *FooDB* (Wishart, 2014), *Toxins and Toxin-Targets Database* (Wishart et al., 2014), *International Classifications of Disease (ICD-11) by WHO* (World Health Organization, 2018), *Metabolic Pathways and Enzymes Database* (Caspi et al., 2017), *Human Metaboleme Database* (Jewell et al., 2007), and USDA food and nutrients database (Haytowitz and Pehrsson, 2018). The majority of these has been used by Liu et al. (2015) to compile their thesauri and databases.

Databases for known Entity-Relations in Biomedical research include DISEASES (Pletscher-Frankild et al., 2015) and DisGeNet (Bauer-Mehren et al., 2010) providing gene-disease relations, CTD (Davis et al., 2012) with relations between chemicals, genes, phenotypes, diseases, exposures and pathways, SIDER (Kuhn et al., 2015) providing drug-side effect relations, STRING (Szklarczyk et al., 2014) with protein-protein interactions, ChemProt (Kringelum et al., 2016) with chemical-protein interactions and PharmGKB (Hewett et al., 2002) providing drug-gene relations. These databases have been used by various authors to evaluate relation extraction systems.

In Table 3, we provide an overview of BioNER tools that are available for different programming languages. While there are several other tools, our selection criterion was to cover the earliest successful implementations, benchmark tools as well as the most recent tools using novel approaches.

**Table 3:** An overview of approaches for BioNER tools.

**NER system**	**References**	**Entity type**	**Learning model**	**Feature model**	**Software**
OGER++	Furrer et al., 2019	Multiple	Hybrid (Dictionary/FFNN)	Rich Features/ word2vec	Python
HUNER	Weber et al., 2019	GE, PR, CH, DI, SP, CL	Machine Learning (LSTM-CRF)	Word2vec	Python
LSTMVoter	Hemati and Mehler, 2019	CH	Machine Learning (Bi-LSTM-CRF)	Character Level features	Python
CollaboNet	Yoon et al., 2019	CH, DI, GE, PR	Machine Learning (Bi-LSTM-CRF)	Character Level WE	Python
MetaMap	Demner-Fushman et al., 2017	Multiple UMLS terms	Dictionary	Tokens/POS	Java
TaggerOne	Leaman and Lu, 2016	DI, CH	Machine Learning (Semi-Markov)	Rich Features	Java
BEST	Lee et al., 2016	Multiple	Dictionary	Tokens/POS	Java
GNormPlus	Wei et al., 2015	GE, PR	Machine Learning (CRF)	Rich Features	Java/Perl
tmChem	Leaman et al., 2015	CH	Machine Learning (Ensemble CRF)	Rich Features	Java/Perl /C++
Dnorm	Leaman et al., 2013	DI	Hybrid (Dictionary/CRF)	Rich Features	Java
ChemSpot	Rocktäschel et al., 2012	CH	Hybrid (CRF/Dictionary)	Rich Fetures	Java
SR4GN	Wei et al., 2012	SP	Hybrid (Dictionary/Rules)	Rich features	Perl
OrganismTagger	Naderi et al., 2011	Genus, SP, Strain	Hybrid (Rule/SVM)	Rich features/ Tokens	Python
Gimli	Campos et al., 2013	PR, DNA, RNA, CL, CT	Hybrid
(Dictionary/CRF)	Rich Features	Java
LINNAEUS	Gerner et al., 2010	SP	Dictionary	Tokens/ Orthographic	Java
BANNER	Leaman and Gonzalez, 2008	DI, GE, PR	Machine Learning (CRF)	Rich features	Java

*GE, genes; PR, proteins; CH, chemicals; DI, diseases; SP, species; CL, cell line; CT, cell type*.

The improvement of resources and techniques for biomedical annotation has also brought about an abundance of open source tools that have simplified the information extraction for relation mining in biomedical texts. Many of these are general-purpose text mining tools that can be easily configured to process biomedical texts. For instance, Xing et al. (2018) used the open information extraction tool OLLIE (Schmitz et al., 2012) to identify relations between genes and phenotypes, whereas (Kim et al., 2017) identified gene-disease relations utilizing DigSee (Kim et al., 2013). Other useful tools that can be application adaptable and have higher F-score measures are; DeepDive (Niu et al., 2012): an information extraction system developed for structuring unstructured text documents, RelEx (Fundel et al., 2006): a dependency parsing based relations extractor applicable to biomedical free text, and PKDE4J (Song et al., 2015): an extractor that combines rule-based and dictionary-based approaches for multiple-entity relation extraction.

Furthermore, there are general NLP tools heavily used in BioNER and BioRD alike for pre-processing and syntactic analysis. These include Stanford CoreNLP (Manning et al., 2014) for general pre-processing, Stanford POS Tagger (Toutanova et al., 2003) and Stanford dependency parser (Chen and Manning, 2014) for syntactic and semantic sentence analysis, Splitta (Gillick, 2009) for sentence splitting, GENIA tagger (Tsuruoka et al., 2005) for POS tagging and semantic analysis and Verbnet (Palmer et al., 2017) for verb extraction.

## 7. Applications

One of the most important applications of BioNER and BioRD is narrowing down the search space when exploring millions of online biomedical journal articles. Often, one needs to find articles that do not merely include a search term but also include contextual information. For example, if “sequenced genes in chromosome 9” is the query, all the articles that contain different gene names should also appear in the search results. That would only be possible if the search method knows how to locate genes as well as classify them as chromosome 9 related.

Another application is for disease diagnosis and treatment, where mining prior treatment data and research work could assist in narrowing down the diagnosis and possibly effective treatment regiments for a given complicated set of symptoms presented by a patient (Zhu et al., 2013; Bello et al., 2019). In recent years, there has been much attention to designing automated healthcare chatbot systems that are configured to respond to user queries and provide advice or support. Healthcare chatbots use various biomedical text mining techniques to process queries, match them to answers in their knowledge base to either provide medical advice or to refer them (Chawla and Anuradha, 2018; Ghosh et al., 2018). Such systems require the ability to process entities and relations such as diseases, drugs, symptoms, body parts, diagnosis, treatments, or adverse effects (Ghiasvand and Kate, 2018; Wang et al., 2018c).

Another notable application of relation detection is for generating biological interaction networks Azam et al. (2019). For instance, a query like “all drugs associated with prostate cancer treatment” requires knowing which tokens refer to drugs and which phrases point to prostate cancer treatment. Once such associations are established, they can be summarized as a network representing prostate cancer gene interactions or drug-to-drug interactions with side effects. These networks not only provide a summation of thousands of research articles and a visualization but also allow us to derive novel hypotheses.

Furthermore, relation extraction can be a vital tool in Adverse Drug Reaction (ADR) and Drug-Drug Interaction (DDI) analysis. It is not practical and ethical to conduct drug trials in a way that all possible DDIs and ADRs are discovered. As such, creating a network with known interactions extracted from research would allow us to explore other possible interactions between drugs and adverse effects Luo et al. (2016).

## 8. Discussion

From a general point of view, the task of performing Named Entity Recognition (NER) and Relation Detection (RD) are data science problems (Emmert-Streib and Dehmer, 2019a). That means an optimal combination of data and methods is required for achieving the best results. Regarding the data, most current studies are based on information from abstracts of scientific articles as provided, e.g., by PubMed. However, such articles contain much more information, which is only accessible if one would have access to full-text publications. For journals having an open access policy like PLoS, Frontiers, or MDPI, this does not constitute an obstacle. However, many articles are still hidden behind a paywall, e.g., most articles from Nature and Science. A related problem refers to capturing information from tables or Supplementary Files. Especially the latter possess new challenges because most publishers do not provide formatting guidelines for Supplementary Files rendering such texts as unstructured. Importantly, information extracted from such full-text publications or Supplementary Files could not only lead to additional information but to redundant information that could be utilized for correcting errors obtained from using journal abstracts solely. Hence, one could expect to improve the quality of the analysis performance by using additional input texts as provided by full-text publications or Supplementary Files. Another problem relates to the extraction of italicized or quoted text which may not be captured.

A common question asked is what is the performance of a method and how does it compare to other related methods? Since the papers reviewed in this article have all been published in either scientific journals or conferences or preprint servers all of them have been studied numerically, at least to some extend. However, for any serious application the information required is the generalization error (GenErr) Emmert-Streib and Dehmer (2019b) and the dependence of the GenErr on variations of the data. The statistical estimation of the GenErr is in general challenging and not straight forward. This implies that this error may be considerably different to the numerical results provided in the reviewed papers and, hence, a case-by-case analysis is required to select the proper method for a given application domain. For this reason, as a warning, we would like to remark that despite the fact that we provided throughout the paper information about obtained F-scores or recall values, such information needs to be considered cautiously. Hence, such values should not be seen as an absolute indicator of performance but as guideline for your own dedicated context-specific performance analysis.

From a methodological point of view, deep learning approaches are still relatively new, leaving plenty of room for improvement (Yadav and Bethard, 2019). A general reason for the popularity of these methods is that deep learning neural networks require no/little feature selection but perform such a mechanism internally within their hidden layers. Certainly, this characteristic is not entirely domain and data-independent (Smolander et al., 2019), and it remains to be seen if this also holds for text data, especially when the number of samples is not in the millions. Interestingly, recent results for patient phenotyping from electronic health records (eHRs) show that this might be the case (Yang et al., 2019). Regarding methods, unsupervised and semi-supervised methods have the most significant potential for improvement because annotated benchmark corpora are still relatively small; see Table 1 and the information about the available sample sizes. Hence, methods that operate, at least partially, unsupervised would be very beneficial because they do not require such annotations yet can harvest from the millions of available publications. This could also be connected to learning representations of sentences or words. A good example of this direction is an extension of BERT (Devlin et al., 2019), where unsupervised pre-training with large-scale biomedical corpora is used followed by task-specific fine tuning. The resulting method called BioBERT (Lee et al., 2019) has been shown to result in state-of-the-art performance in a number of different biomedical tasks, including biomedical named entity recognition, biomedical relation extraction and biomedical question answering.

Looking back, methods for word embedding made tremendous progress in recent years starting with word2vec and the improvement by BERT. These results have been enabled by exploiting different neural network architectures (e.g., bidirectional transformers for BERT and LSTMs for ELMo). It seems natural to further explore this direction, e.g., by using nested architectures or introducing additional training or pre-training steps for combined network architectures.

Related to the last point above is learning new sentence representations in the form of trees or general graphs (Luo et al., 2016). A potential advantage of such a representation is that the rich information from network studies about graph energy, graph entropy, molecular descriptors, or network comparisons could be utilized (Todeschini et al., 2002; Li et al., 2012; Dehmer et al., 2016; Emmert-Streib et al., 2016). For instance, starting from a dependency parse, refined representations could be learned using unsupervised approaches, e.g., autoencoders, to enhance the captured features (Eisenstein, 2019). Importantly, not only deep learning methods might be relevant but also SVMs by deriving new graph kernels from such refined graph representations and, e.g., graph descriptors (Vishwanathan et al., 2010; Panyam et al., 2018b). Furthermore, we would like to note that NLP methods can contain subjective notions. For instance, for a polarity analysis there is no objective way to derive the meaning of a “positive” or “negative” association. Instead, this information needs to be defined by the user. Hence, such an analysis captures the definition of the user.

Finally, another recent development is provided by end-to-end learning Li and Ji (2014). For end-to-end learning the NER and RD tasks are jointly learned, as opposed to pipeline-based systems, because this has been shown to minimize error propagation and improve performance (Giorgi et al., 2019). Generally, end-to-end systems can be either trained as a sequentially (Li et al., 2015a,b; Bekoulis et al., 2018a) or as a simultaneous learning process for both NER and RD. The latter approach is more recent, yet successful with state-of-the-art performance (Li et al., 2017a; Bekoulis et al., 2018b). While it is common for most end-to-end approaches to get some help from external NLP tools for auxiliary tasks, e.g., dependency parsers, Giorgi et al. (2019) proposed a model to be truly end-to-end with no external help. Problems current systems struggle with are nested entities and inter-sentence relations. Both issues provide ample opportunities for future research.

## 9. Conclusion

In this paper, we reviewed methods for Named Entity Recognition (NER) and Relation Detection (RD) allowing, e.g., to identify interactions between proteins and drugs or genes and diseases. Over the years, many methods have been introduced and studied for resolving a variety of problems in biomedical, health, and clinical sciences. For this reason, we aimed for a systematic presentation by categorizing methods according to their main characteristics. Importantly, recent progress in artificial intelligence via deep learning provided a new perspective on NER and RD, and further advances can be expected in this direction in the near future.

## Author Contributions

FE-S conceived the study. All authors wrote the article and approved the final version.

## Conflict of Interest

The authors declare that the research was conducted in the absence of any commercial or financial relationships that could be construed as a potential conflict of interest.

## References

[B1] Amberger J. S. Bocchini C. A. Scott A. F. Hamosh A. (2018). Omim. org: leveraging knowledge across phenotype-gene relationships. Nucleic Acids Res. 47, D1038–D1043. 10.1093/nar/gky1151 30445645 PMC6323937

[B2] Azam M. Musa A. Dehmer M. Yli-Harja O. Emmert-Streib F. (2019). Global genetics research in prostate cancer: a text mining and computational network theory approach. Front. Genet. 10:70. 10.3389/fgene.2019.00070 30838019 PMC6383410

[B3] Bach N. Badaskar S. (2007). A review of relation extraction. Literature review for Language and Statistics II 2. Available online at: https://www.researchgate.net/profile/Nguyen_Bach3/publication/265006408_A_Review_of_Relation_Extraction/links/54cacfe70cf2c70ce52401c9/A-Review-of-Relation-Extraction.pdf

[B4] Bada M. Eckert M. Evans D. Garcia K. Shipley K. Sitnikov D.. (2012). Concept annotation in the craft corpus. BMC Bioinform. 13:161. 10.1186/1471-2105-13-161 22776079 PMC3476437

[B5] Bastian M. Heymann S. Jacomy M. (2009). Gephi: an open source software for exploring and manipulating networks, in Third International AAAI Conference on Weblogs and Social Media.

[B6] Bauer-Mehren A. Rautschka M. Sanz F. Furlong L. I. (2010). Disgenet: a cytoscape plugin to visualize, integrate, search and analyze gene-disease networks. Bioinformatics 26, 2924–2926. 10.1093/bioinformatics/btq538 20861032

[B7] Bekoulis G. Deleu J. Demeester T. Develder C. (2018a). Adversarial training for multi-context joint entity and relation extraction. arXiv [Preprint]. arXiv:1808.06876. 10.18653/v1/D18-1307

[B8] Bekoulis G. Deleu J. Demeester T. Develder C. (2018b). Joint entity recognition and relation extraction as a multi-head selection problem. Expert Syst. Appl. 114, 34–45. 10.1016/j.eswa.2018.07.032

[B9] Bell D. Hahn-Powell G. Valenzuela-Escárcega M. A. Surdeanu M. (2016). Sieve-based coreference resolution in the biomedical domain. arXiv [Preprint]. arXiv:1603.03758.

[B10] Bello F. L. Naya H. Raggio V. Rosá A. (2019). From medical records to research papers: a literature analysis pipeline for supporting medical genomic diagnosis processes. Inform. Med. Unlocked 15:100181 10.1016/j.imu.2019.100181

[B11] Bengio Y. Ducharme R. Vincent P. Jauvin C. (2003). A neural probabilistic language model. J. Mach. Learn. Res. 3, 1137–1155. Available online at: http://www.jmlr.org/papers/volume3/bengio03a/bengio03a.pdf18390314

[B12] Bethesda N. U. (2005). Pubmed help.

[B13] Bethesda N. U. (2019). Medline: description of the database. Available online at: https://www.nlm.nih.gov/bsd/medline.html

[B14] Bhasuran B. Murugesan G. Abdulkadhar S. Natarajan J. (2016). Stacked ensemble combined with fuzzy matching for biomedical named entity recognition of diseases. J. Biomed. Inform. 64, 1–9. 10.1016/j.jbi.2016.09.009 27634494

[B15] Bhasuran B. Natarajan J. (2018). Automatic extraction of gene-disease associations from literature using joint ensemble learning. PLoS ONE 13:e0200699. 10.1371/journal.pone.0200699 30048465 PMC6061985

[B16] Björne J. Salakoski T. (2018). Biomedical event extraction using convolutional neural networks and dependency parsing, in Proceedings of the BioNLP 2018 Workshop, 98–108. 10.18653/v1/W18-2311

[B17] Braud C. Denis P. (2015). Comparing word representations for implicit discourse relation classification, in Proceedings of the 2015 Conference on Empirical Methods in Natural Language Processing, 2201–2211. 10.18653/v1/D15-1262

[B18] Brown P. F. Desouza P. V. Mercer R. L. Pietra V. J. D. Lai J. C. (1992). Class-based n-gram models of natural language. Comput. Linguist. 18, 467–479.

[B19] Bundschus M. Dejori M. Stetter M. Tresp V. Kriegel H.-P. (2008). Extraction of semantic biomedical relations from text using conditional random fields. BMC Bioinform. 9:207. 10.1186/1471-2105-9-207 18433469 PMC2386138

[B20] Bunescu R. Ge R. Kate R. J. Marcotte E. M. Mooney R. J. Ramani A. K.. (2005). Comparative experiments on learning information extractors for proteins and their interactions. Artif. Intell. Med. 33, 139–155. 10.1016/j.artmed.2004.07.016 15811782

[B21] Campos D. Matos S. Oliveira J. L. (2012). Biomedical named entity recognition: a survey of machine-learning tools. Theory Appl. Adv. Text Mining 175–195. 10.5772/5106627589962

[B22] Campos D. Matos S. Oliveira J. L. (2013). Gimli: open source and high-performance biomedical name recognition. BMC Bioinform. 14:54. 10.1186/1471-2105-14-54 23413997 PMC3651325

[B23] Caspi R. Billington R. Fulcher C. A. Keseler I. M. Kothari A. Krummenacker M.. (2017). The metacyc database of metabolic pathways and enzymes. Nucleic Acids Res. 46, D633–D639. 10.1093/nar/gkx935 29059334 PMC5753197

[B24] Chawla R. Anuradha J. (2018). Counsellor chatbot. Comput. Sci. 5, 126–136. Available online at: https://www.academia.edu/36353256/COUNSELLOR_CHATBOT

[B25] Chen D. Manning C. (2014). A fast and accurate dependency parser using neural networks, in Proceedings of the 2014 Conference on Empirical Methods in Natural Language Processing (EMNLP), 740–750. 10.3115/v1/D14-1082

[B26] Cheng D. Knox C. Young N. Stothard P. Damaraju S. Wishart D. S. (2008). Polysearch: a web-based text mining system for extracting relationships between human diseases, genes, mutations, drugs and metabolites. Nucleic Acids Res. 36, W399–W405. 10.1093/nar/gkn296 18487273 PMC2447794

[B27] Cohen K. B. Lanfranchi A. Choi M. J.-y. Bada M. Baumgartner W. A. Panteleyeva N.. (2017). Coreference annotation and resolution in the colorado richly annotated full text (craft) corpus of biomedical journal articles. BMC Bioinform. 18:372. 10.1186/s12859-017-1775-9 28818042 PMC5561560

[B28] Collobert R. Weston J. (2008). A unified architecture for natural language processing: deep neural networks with multitask learning, in Proceedings of the 25th International Conference on Machine Learning (ACM), 160–167. 10.1145/1390156.1390177

[B29] Collobert R. Weston J. Bottou L. Karlen M. Kavukcuoglu K. Kuksa P. (2011). Natural language processing (almost) from scratch. J. Mach. Learn. Res. 12, 2493–2537. Available online at: http://www.jmlr.org/papers/volume12/collobert11a/collobert11a.pdf

[B30] Consortium G. O. (2004). The gene ontology (go) database and informatics resource. Nucleic Acids Res. 32, D258–D261. 10.1093/nar/gkh036 14681407 PMC308770

[B31] Coulet A. Shah N. H. Garten Y. Musen M. Altman R. B. (2010). Using text to build semantic networks for pharmacogenomics. J. Biomed. Inform. 43, 1009–1019. 10.1016/j.jbi.2010.08.005 20723615 PMC2991587

[B32] Davis A. P. Murphy C. G. Johnson R. Lay J. M. Lennon-Hopkins K. Saraceni-Richards C.. (2012). The comparative toxicogenomics database: update 2013. Nucleic Acids Res. 41, D1104–D1114. 10.1093/nar/gks994 23093600 PMC3531134

[B33] Dehmer M. Emmert-Streib F. Chen Z. Li X. Shi Y. (2016). Mathematical Foundations and Applications of Graph Entropy. Wiley Online Library 10.1002/9783527693245

[B34] Demner-Fushman D. Rogers W. J. Aronson A. R. (2017). Metamap lite: an evaluation of a new java implementation of metamap. J. Am. Med. Inform. Assoc. 24, 841–844. 10.1093/jamia/ocw177 28130331 PMC6080672

[B35] Denecke K. Deng Y. (2015). Sentiment analysis in medical settings: new opportunities and challenges. Artif. Intell. Med. 64, 17–27. denecke2015sentiment. 10.1016/j.artmed.2015.03.006 25982909

[B36] Devlin J. Chang M.-W. Lee K. Toutanova K. (2018). Bert: pre-training of deep bidirectional transformers for language understanding. arXiv [Preprint]. arXiv:1810.04805.

[B37] Devlin J. Chang M.-W. Lee K. Toutanova K. (2019). Bert: pre-training of deep bidirectional transformers for language understanding, in Proceedings of the 2019 Conference of the North American Chapter of the Association for Computational Linguistics: Human Language Technologies, Volume 1 (Long and Short Papers), 4171–4186.

[B38] Dogan R. I. Leaman R. Lu Z. (2014). NCBI disease corpus: a resource for disease name recognition and concept normalization. J. Biomed. Inform. 47, 1–10. 10.1016/j.jbi.2013.12.006 24393765 PMC3951655

[B39] D'Souza J. Ng V. (2012). Anaphora resolution in biomedical literature: a hybrid approach, in Proceedings of the ACM Conference on Bioinformatics, Computational Biology and Biomedicine (ACM), 113–122. 10.1145/2382936.2382951

[B40] Duque A. Stevenson M. Martinez-Romo J. Araujo L. (2018). Co-occurrence graphs for word sense disambiguation in the biomedical domain. Artif. Intell. Med. 87, 9–19. 10.1016/j.artmed.2018.03.002 29573845

[B41] Eftimov T. Seljak B. K. Korošec P. (2017). A rule-based named-entity recognition method for knowledge extraction of evidence-based dietary recommendations. PLoS ONE 12:e0179488. 10.1371/journal.pone.0179488 28644863 PMC5482438

[B42] Eisenstein J. (2019). Introduction to Natural Language Processing. MIT Press.

[B43] Eltyeb S. Salim N. (2014). Chemical named entities recognition: a review on approaches and applications. J. Cheminform. 6:17. 10.1186/1758-2946-6-17 24834132 PMC4022577

[B44] Emmert-Streib F. Dehmer M. (2011). Networks for Systems Biology: Conceptual Connection of Data and Function. IET Syst. Biol. 5:185. 10.1049/iet-syb.2010.0025 21639592

[B45] Emmert-Streib F. Dehmer M. (2019a). Defining data science by a data-driven quantification of the community. Mach. Learn. Knowledge Extract. 1, 235–251. 10.3390/make1010015

[B46] Emmert-Streib F. Dehmer M. (2019b). Evaluation of regression models: model assessment, model selection and generalization error. Mach. Learn. Knowledge Extract. 1, 521–551. 10.3390/make1010032

[B47] Emmert-Streib F. Dehmer M. Shi Y. (2016). Fifty years of graph matching, network alignment and network comparison. Inform. Sci. 346–347, 180–197. 10.1016/j.ins.2016.01.074

[B48] Emmert-Streib F. Moutari S. Dehmer M. (2019). A comprehensive survey of error measures for evaluating binary decision making in data science. Wiley Interdiscipl. Rev. Data Mining Knowledge Discov. e1303. 10.1002/widm.1303 31656552 PMC6777486

[B49] Emmert-Streib F. Musa A. Tripathi S. Yli-Harja O. Baltakys K. Kanniainen J. (2018). Computational analysis of structural properties of economic networks. J. Netw. Theory Fin. 4, 1–32. 10.21314/JNTF.2018.043

[B50] Emmert-Streib F. Yang Z. Feng H. Tripathi S. Dehmer M. (2020). An introductory review of deep learning for prediction models with big data. Front. Artif. Intell. 3:4 10.3389/frai.2020.00004 PMC7861305 33733124

[B51] Franz M. Lopes C. T. Huck G. Dong Y. Sumer O. Bader G. D. (2015). Cytoscape.js: a graph theory library for visualisation and analysis. Bioinformatics 32, 309–311. Available online at: https://academic.oup.com/bioinformatics/article/32/2/309/1744007 26415722 10.1093/bioinformatics/btv557 PMC4708103

[B52] Fundel K. Küffner R. Zimmer R. (2006). Relex-relation extraction using dependency parse trees. Bioinformatics 23, 365–371. 10.1093/bioinformatics/btl616 17142812

[B53] Furrer L. Jancso A. Colic N. Rinaldi F. (2019). Oger++: hybrid multi-type entity recognition. J. Cheminform. 11:7. 10.1186/s13321-018-0326-3 30666476 PMC6689863

[B54] Gaizauskas R. Demetriou G. Artymiuk P. J. Willett P. (2003). Protein structures and information extraction from biological texts: the pasta system. Bioinformatics 19, 135–143. 10.1093/bioinformatics/19.1.135 12499303

[B55] Gaudan S. Kirsch H. Rebholz-Schuhmann D. (2005). Resolving abbreviations to their senses in Medline. Bioinformatics 21, 3658–3664. 10.1093/bioinformatics/bti586 16037121

[B56] Gerner M. Nenadic G. Bergman C. M. (2010). Linnaeus: a species name identification system for biomedical literature. BMC Bioinform. 11:85. 10.1186/1471-2105-11-85 20149233 PMC2836304

[B57] Ghiasvand O. Kate R. J. (2018). Learning for clinical named entity recognition without manual annotations. Inform. Med. Unlocked 13, 122–127. 10.1016/j.imu.2018.10.011

[B58] Ghosh S. Bhatia S. Bhatia A. (2018). Quro: facilitating user symptom check using a personalised chatbot-oriented dialogue system. Stud. Health Technol. Inform. 252:51. 30040682

[B59] Gillick D. (2009). Sentence boundary detection and the problem with the us, in Proceedings of Human Language Technologies: The 2009 Annual Conference of the North American Chapter of the Association for Computational Linguistics, Companion Volume: Short Papers, 241–244. 10.3115/1620853.1620920

[B60] Giorgi J. Bader G. (2019). Towards reliable named entity recognition in the biomedical domain. bioRxiv 526244. 10.1101/526244. Available online at: https://www.intechopen.com/books/theory-and-applications-for-advanced-text-mining/biomedical-named-entity-recognition-a-survey-of-machine-learning-tools 31218364 PMC6956779

[B61] Giorgi J. Wang X. Sahar N. Shin W. Y. Bader G. D. Wang B. (2019). End-to-end named entity recognition and relation extraction using pre-trained language models. arXiv [Preprint]. arXiv:1912.13415.

[B62] Goyal A. Gupta V. Kumar M. (2018). Recent named entity recognition and classification techniques: a systematic review. Comput. Sci. Rev. 29, 21–43. goyal2018recent. 10.1016/j.cosrev.2018.06.001

[B63] Habibi M. Weber L. Neves M. Wiegandt D. L. Leser U. (2017). Deep learning with word embeddings improves biomedical named entity recognition. Bioinformatics 33, i37–i48. 10.1093/bioinformatics/btx228 28881963 PMC5870729

[B64] Haytowitz D. B. Pehrsson P. R. (2018). USDA'S national food and nutrient analysis program (NFNAP) produces high-quality data for USDA food composition databases: two decades of collaboration. Food Chem. 238, 134–138. 10.1016/j.foodchem.2016.11.082 28867083

[B65] Hemati W. Mehler A. (2019). LSTMVoter: chemical named entity recognition using a conglomerate of sequence labeling tools. J. Cheminform. 11:3. 10.1186/s13321-018-0327-2 30631966 PMC6689880

[B66] Herrero-Zazo M. Segura-Bedmar I. Martínez P. Declerck T. (2013). The DDI corpus: an annotated corpus with pharmacological substances and drug-drug interactions. J. Biomed. Inform. 46, 914–920. 10.1016/j.jbi.2013.07.011 23906817

[B67] Hewett M. Oliver D. E. Rubin D. L. Easton K. L. Stuart J. M. Altman R. B.. (2002). PharmGKB: the pharmacogenetics knowledge base. Nucleic Acids Res. 30, 163–165. 10.1093/nar/30.1.163 11752281 PMC99138

[B68] Hsieh Y.-L. Chang Y.-C. Chang N.-W. Hsu W.-L. (2017). Identifying protein-protein interactions in biomedical literature using recurrent neural networks with long short-term memory, in Proceedings of the Eighth International Joint Conference on Natural Language Processing (Volume 2: Short Papers), 240–245. hsieh2017identifying.

[B69] Hua L. Quan C. (2016a). A shortest dependency path based convolutional neural network for protein-protein relation extraction. BioMed research international 2016. depend2. 10.1155/2016/8479587 27493967 PMC4963603

[B70] Hua L. Quan C. (2016b). A shortest dependency path based convolutional neural network for protein-protein relation extraction. BioMed Res. Int. 2016:8479587. 27493967 10.1155/2016/8479587 PMC4963603

[B71] Huang M.-S. Lai P.-T. Tsai R. T.-H. Hsu W.-L. (2019). Revised jnlpba corpus: a revised version of biomedical ner corpus for relation extraction task. arXiv [Preprint]. arXiv:1901.10219.

[B72] Intxaurrondo A. Pérez-Pérez M. Pérez-Rodríguez G. López-Martín J. A. Santamaria J. de la Pena S. (2017). The biomedical abbreviation recognition and resolution (barr) track: benchmarking, evaluation and importance of abbreviation recognition systems applied to spanish biomedical abstracts. Available online at: https://upcommons.upc.edu/handle/2117/107342

[B73] Ion R. (2007). TTL: A Portable Framework for Tokenization, Tagging and Lemmatization of Large Corpora. Bucharest: Romanian Academy.

[B74] Jensen K. Panagiotou G. Kouskoumvekaki I. (2014). Integrated text mining and chemoinformatics analysis associates diet to health benefit at molecular level. PLoS Comput. Biol. 10:e1003432. 10.1371/journal.pcbi.1003432 24453957 PMC3894162

[B75] Jettakul A. Wichadakul D. Vateekul P. (2019). Relation extraction between bacteria and biotopes from biomedical texts with attention mechanisms and domain-specific contextual representations. BMC Bioinformatics 20:627. 10.1186/s12859-019-3217-3 31795930 PMC6889521

[B76] Jewell K. Arndt D. Sawhney S. Fung C. Nikolai L. Lewis M.. (2007). HMDB: the human metabolome database. Nucleic Acids Res. 35. 10.1093/nar/gkl923. Available online at: https://www.hindawi.com/journals/bmri/2015/918710/17202168 PMC1899095

[B77] Jing K. Xu J. He B. (2019). A survey on neural network language models. arXiv [Preprint]. arXiv:1906.03591.

[B78] Joulin A. Grave E. Bojanowski P. Douze M. Jégou H. Mikolov T. (2016). Fasttext. zip: compressing text classification models. arXiv [Preprint]. arXiv:1612.03651.

[B79] Jovanović J. Bagheri E. (2017). Semantic annotation in biomedicine: the current landscape. J. Biomed. Semant. 8:44. 10.1186/s13326-017-0153-x 28938912 PMC5610427

[B80] Kazama J. Makino T. Ohta Y. Tsujii J. (2002). Tuning support vector machines for biomedical named entity recognition, in Proceedings of the ACL-02 Workshop on Natural Language Processing in the Biomedical Domain, Vol. 3 (Association for Computational Linguistics), 1–8. 10.3115/1118149.1118150

[B81] Keretna S. Lim C. P. Creighton D. Shaban K. B. (2015). Enhancing medical named entity recognition with an extended segment representation technique. Comput. Methods Prog. Biomed. 119, 88–100. 10.1016/j.cmpb.2015.02.007 25791277

[B82] Keshava Prasad T. Goel R. Kandasamy K. Keerthikumar S. Kumar S. Mathivanan S.. (2008). Human protein reference database-2009 update. Nucleic Acids Res. 37, D767–D772. 10.1093/nar/gkn892 18988627 PMC2686490

[B83] Kilicoglu H. Bergler S. (2009). Syntactic dependency based heuristics for biological event extraction, in Proceedings of the BioNLP 2009 Workshop Companion Volume for Shared Task, 119–127. 10.3115/1572340.1572361

[B84] Kim J. Kim J.-J. Lee H. (2017). An analysis of disease-gene relationship from medline abstracts by digsee. Sci. Rep. 7:40154 10.1038/srep40154 28054646 PMC5215527

[B85] Kim J. So S. Lee H.-J. Park J. C. Kim J.-j. Lee H. (2013). DIGSEE: disease gene search engine with evidence sentences (version cancer). Nucleic Acids Res. 41, W510–517. 10.1093/nar/gkt531 23761452 PMC3692119

[B86] Kim J.-D. Ohta T. Pyysalo S. Kano Y. Tsujii J. (2009). Overview of bioNLP'09 shared task on event extraction, in Proceedings of the Workshop on Current Trends in Biomedical Natural Language Processing: Shared Task (Association for Computational Linguistics), 1–9. 10.3115/1572340.1572342

[B87] Kim J.-D. Ohta T. Tateisi Y. Tsujii J. (2003). Genia corpus- A semantically annotated corpus for bio-textmining. Bioinformatics 19, i180–182. 10.1093/bioinformatics/btg1023 12855455

[B88] Kim S. Liu H. Yeganova L. Wilbur W. J. (2015). Extracting drug-drug interactions from literature using a rich feature-based linear kernel approach. J. Biomed. Inform. 55, 23–30. 10.1016/j.jbi.2015.03.002 25796456 PMC4464931

[B89] Kim Y. Jernite Y. Sontag D. Rush A. M. (2016). Character-aware neural language models, in Thirtieth AAAI Conference on Artificial Intelligence.

[B90] Kipf T. N. Welling M. (2016). Semi-supervised classification with graph convolutional networks. arXiv [Preprint]. arXiv:1609.02907.

[B91] Kolchinsky A. Lourenço A. Wu H.-Y. Li L. Rocha L. M. (2015). Extraction of pharmacokinetic evidence of drug-drug interactions from the literature. PLoS ONE 10:e0122199. 10.1371/journal.pone.0122199 25961290 PMC4427505

[B92] Krallinger M. Leitner F. Rabal O. Vazquez M. Oyarzabal J. Valencia A. (2015). CHEMDNER: the drugs and chemical names extraction challenge. J. Cheminform. 7:S1. 10.1186/1758-2946-7-S1-S1 25810766 PMC4331685

[B93] Krallinger M. Leitner F. Rodriguez-Penagos C. Valencia A. (2008). Overview of the protein-protein interaction annotation extraction task of biocreative II. Genome Biol. 9:S4. 10.1186/gb-2008-9-s2-s4 18834495 PMC2559988

[B94] Krallinger M. Rabal O. Akhondi S. A. Pérez MP Santamaria JL Rodriguez GP (2017). Overview of the biocreative VI chemical-protein interaction track, in Proceedings of the Sixth BioCreative Challenge Evaluation Workshop, 141–146.

[B95] Kringelum J. Kjaerulff S. K. Brunak S. Lund O. Oprea T. I. Taboureau O. (2016). Chemprot-3.0: a global chemical biology diseases mapping. Database 2016. 10.1093/database/bav123 26876982 PMC4752971

[B96] Kuhn M. Letunic I. Jensen L. J. Bork P. (2015). The sider database of drugs and side effects. Nucleic Acids Res. 44, D1075–D1079. 10.1093/nar/gkv1075 26481350 PMC4702794

[B97] Lafferty J. McCallum A. Pereira F. C. (2001). Conditional random fields: probabilistic models for segmenting and labeling sequence data. Available online at: https://repository.upenn.edu/cis_papers/159/

[B98] Leaman R. Gonzalez G. (2008). Banner: an executable survey of advances in biomedical named entity recognition, in Biocomputing 2008 (World Scientific), 652–663. 10.1142/9789812776136_0062 18229723

[B99] Leaman R. Islamaj Doğan R. Lu Z. (2013). DNorm: disease name normalization with pairwise learning to rank. Bioinformatics 29, 2909–2917. 10.1093/bioinformatics/btt474 23969135 PMC3810844

[B100] Leaman R. Lu Z. (2016). TaggerOne: joint named entity recognition and normalization with semi-Markov models. Bioinformatics 32, 2839–2846. 10.1093/bioinformatics/btw343 27283952 PMC5018376

[B101] Leaman R. Wei C.-H. Lu Z. (2015). TMChem: a high performance approach for chemical named entity recognition and normalization. J. Cheminform. 7:S3. 10.1186/1758-2946-7-S1-S3 25810774 PMC4331693

[B102] LeCun Y. Bengio Y. Hinton G. (2015). Deep learning. Nature 521:436. 10.1038/nature14539 26017442

[B103] Lee J. Yoon W. Kim S. Kim D. Kim S. So C. H.. (2019). BioBERT: a pre-trained biomedical language representation model for biomedical text mining. Bioinformatics. 10.1093/bioinformatics/btz682 31501885 PMC7703786

[B104] Lee J. Yoon W. Kim S. Kim D. Kim S. So C. H.. (2020). BioBERT: a pre-trained biomedical language representation model for biomedical text mining. Bioinformatics 36, 1234–1240. 31501885 10.1093/bioinformatics/btz682 PMC7703786

[B105] Lee K. He L. Lewis M. Zettlemoyer L. (2017). End-to-end neural coreference resolution. arXiv [Preprint]. arXiv:1707.07045. 10.18653/v1/D17-1018

[B106] Lee S. Kim D. Lee K. Choi J. Kim S. Jeon M.. (2016). Best: next-generation biomedical entity search tool for knowledge discovery from biomedical literature. PLoS ONE 11:e0164680. 10.1371/journal.pone.0164680 27760149 PMC5070740

[B107] Leitner F. Mardis S. A. Krallinger M. Cesareni G. Hirschman L. A. Valencia A. (2010). An overview of biocreative II. 5. IEEE/ACM Trans. Comput. Biol. Bioinform. 7, 385–399. 10.1109/TCBB.2010.61 20704011

[B108] Leser U. Hakenberg J. (2005). What makes a gene name? Named entity recognition in the biomedical literature. Brief. Bioinform. 6, 357–369. 10.1093/bib/6.4.357 16420734

[B109] Levy O. Goldberg Y. (2014). Dependency-based word embeddings, in Proceedings of the 52nd Annual Meeting of the Association for Computational Linguistics (Volume 2: Short Papers), 302–308. 10.3115/v1/P14-2050

[B110] Li C. Liakata M. Rebholz-Schuhmann D. (2013). Biological network extraction from scientific literature: state of the art and challenges. Brief. Bioinform. 15, 856–877. 10.1093/bib/bbt006 23434632

[B111] Li C. Liakata M. Rebholz-Schuhmann D. (2014). Biological network extraction from scientific literature: state of the art and challenges. Brief. Bioinform. 15, 856–877. 23434632 10.1093/bib/bbt006

[B112] Li F. Zhang M. Fu G. Ji D. (2017a). A neural joint model for entity and relation extraction from biomedical text. BMC Bioinform. 18, 1–11. 10.1186/s12859-017-1609-9 28359255 PMC5374588

[B113] Li G. Ross K. E. Arighi C. N. Peng Y. Wu C. H. Vijay-Shanker K. (2015a). miRTEX: a text mining system for miRNA-gene relation extraction. PLoS Comput. Biol. 11:e1004391. 10.1371/journal.pcbi.1004391 26407127 PMC4583433

[B114] Li H. Chen Q. Chen K. Tang B. (2015b). HITSZ_CDR system for disease and chemical named entity recognition and relation extraction, in Proceedings of the Fifth BioCreative Challenge Evaluation Workshop, 196–201. 27270713

[B115] Li H. Chen Q. Tang B. Wang X. Xu H. Wang B.. (2017b). CNN-based ranking for biomedical entity normalization. BMC Bioinformatics 18:385. 10.1186/s12859-017-1805-7 28984180 PMC5629610

[B116] Li J. Sun Y. Johnson R. J. Sciaky D. Wei C.-H. Leaman R.. (2016a). Biocreative V CDR task corpus: a resource for chemical disease relation extraction. Database 2016. 10.1093/database/baw068 PMC4860626 27161011

[B117] Li L. Jin L. Huang D. (2015c). Exploring recurrent neural networks to detect named entities from biomedical text, in Chinese Computational Linguistics and Natural Language Processing Based on Naturally Annotated Big Data (Springer), 279–290. 10.1007/978-3-319-25816-4_23

[B118] Li L. Jin L. Jiang Y. Huang D. (2016b). Recognizing biomedical named entities based on the sentence vector/twin word embeddings conditioned bidirectional LSTM, in Chinese Computational Linguistics and Natural Language Processing Based on Naturally Annotated Big Data (Springer), 165–176. 10.1007/978-3-319-47674-2_15

[B119] Li Q. Ji H. (2014). Incremental joint extraction of entity mentions and relations, in Proceedings of the 52nd Annual Meeting of the Association for Computational Linguistics (Volume 1: Long Papers), 402–412. 10.3115/v1/P14-1038

[B120] Li X. Shi Y. Gutman I. (2012). Graph Energy. Springer Science & Business Media. 10.1007/978-1-4614-4220-2

[B121] Li Z. Yang Z. Shen C. Xu J. Zhang Y. Xu H. (2019). Integrating shortest dependency path and sentence sequence into a deep learning framework for relation extraction in clinical text. BMC Med. Informatics Decis. Mak. 19:22. 10.1186/s12911-019-0736-9 30700301 PMC6354333

[B122] Ling Y. Hasan S. A. Farri O. Chen Z. van Ommering R. Yee C.. (2019). A domain knowledge-enhanced LSTM-CRF model for disease named entity recognition. AMIA Summits Transl. Sci. Proc. 2019:761. 31259033 PMC6568095

[B123] Liu H. Hunter L. Kešelj V. Verspoor K. (2013). Approximate subgraph matching-based literature mining for biomedical events and relations. PLoS ONE 8:e60954. 10.1371/journal.pone.0060954 23613763 PMC3629260

[B124] Liu S. Tang B. Chen Q. Wang X. (2016). Drug-drug interaction extraction via convolutional neural networks. Comput. Math. Methods Med. 2016. 10.1155/2016/6918381 26941831 PMC4752975

[B125] Liu Y. Liang Y. Wishart D. (2015). Polysearch2: a significantly improved text-mining system for discovering associations between human diseases, genes, drugs, metabolites, toxins and more. Nucleic Acids Res. 43, W535-W542. 10.1093/nar/gkv383 25925572 PMC4489268

[B126] Luo Y. Uzuner Ö. Szolovits P. (2016). Bridging semantics and syntax with graph algorithms- State-of-the-art of extracting biomedical relations. Brief. Bioinform. 18, 160–178. 10.1093/bib/bbw001 26851224 PMC5221425

[B127] MacKinlay A. Martinez D. Yepes A. J. Liu H. Wilbur W. J. Verspoor K. (2013). Extracting biomedical events and modifications using subgraph matching with noisy training data, in Proceedings of the BioNLP Shared Task 2013 Workshop, 35–44.

[B128] Mallory E. K. Zhang C. Ré C. Altman R. B. (2015). Large-scale extraction of gene interactions from full-text literature using deepdive. Bioinformatics 32, 106–113. 10.1093/bioinformatics/btv476 26338771 PMC4681986

[B129] Manning C. Surdeanu M. Bauer J. Finkel J. Bethard S. McClosky D. (2014). The Stanford coreNLP natural language processing toolkit, in Proceedings of 52nd Annual Meeting of the Association for Computational Linguistics: System Demonstrations, 55–60.

[B130] Mansouri A. Affendey L. S. Mamat A. (2008). Named entity recognition approaches. Int. J. Comput. Sci. Netw. Secur. 8, 339–344.

[B131] Marrero M. Urbano J. Sánchez-Cuadrado S. Morato J. Gómez-Berbís J. M. (2013). Named entity recognition: fallacies, challenges and opportunities. Comput. Standards Interfaces 35, 482–489. 10.1016/j.csi.2012.09.004

[B132] Miao Q. Zhang S. Meng Y. Fu Y. Yu H. (2012a). Healthy or harmful? Polarity analysis applied to biomedical entity relationships, in Pacific Rim International Conference on Artificial Intelligence (Springer), 777–782. 10.1007/978-3-642-32695-0_72

[B133] Miao Q. Zhang S. Meng Y. Yu H. (2012b). Polarity analysis for food and disease relationships, in Proceedings of the 2012 IEEE/WIC/ACM International Joint Conferences on Web Intelligence and Intelligent Agent Technology (IEEE Computer Society), 188–195. 10.1109/WI-IAT.2012.14

[B134] Mikolov T. Chen K. Corrado G. Dean J. (2013a). Efficient estimation of word representations in vector space. arXiv [Preprint]. arXiv:1301.3781.

[B135] Mikolov T. Sutskever I. Chen K. Corrado G. S. Dean J. (2013b). Distributed representations of words and phrases and their compositionality, in Advances in Neural Information Processing Systems, 3111–3119. Available online at: https://link.springer.com/referenceworkentry/10.1007%2F978-1-4419-9863-7_151

[B136] Miner G. Elder J. IV. Fast A. Hill T. Nisbet R. Delen D. (2012). Practical Text Mining and Statistical Analysis for Non-Structured Text Data Applications. Academic Press.

[B137] Mitrofan M. Ion R. (2017). Adapting the TTL Romanian POS tagger to the biomedical domain, in BiomedicalNLP@ RANLP, 8–14. 10.26615/978-954-452-044-1_002

[B138] Munkhdalai T. Li M. Batsuren K. Park H. A. Choi N. H. Ryu K. H. (2015). Incorporating domain knowledge in chemical and biomedical named entity recognition with word representations. J. Cheminform. 7:S9. 10.1186/1758-2946-7-S1-S9 25810780 PMC4331699

[B139] Nadeau D. Sekine S. (2007). A survey of named entity recognition and classification. Lingvistica Investigationes 30, 3–26. 10.1075/li.30.1.03nad

[B140] Naderi N. Kappler T. Baker C. J. Witte R. (2011). Organismtagger: detection, normalization and grounding of organism entities in biomedical documents. Bioinformatics 27, 2721–2729. 10.1093/bioinformatics/btr452 21828087

[B141] Nayel H. A. Shashirekha H. Shindo H. Matsumoto Y. (2019). Improving multi-word entity recognition for biomedical texts. arXiv [Preprint]. arXiv:1908.05691.

[B142] Niu F. Zhang C. Ré C. Shavlik J. W. (2012). DeepDIVE: Web-scale knowledge-base construction using statistical learning and inference. VLDS 12, 25–28.

[B143] Nobata C. Collier N. Tsujii J.-I. (1999). Automatic term identification and classification in biology texts, in Proc. of the 5th NLPRS, 369–374.

[B144] Nobata C. Dobson P. D. Iqbal S. A. Mendes P. Tsujii J. Kell D. B.. (2011). Mining metabolites: extracting the yeast metabolome from the literature. Metabolomics 7, 94–101. 10.1007/s11306-010-0251-6 21687783 PMC3111869

[B145] Ohta T. Pyysalo S. Tsujii J. Ananiadou S. (2012). Open-domain anatomical entity mention detection, in Proceedings of the Workshop on Detecting Structure in Scholarly Discourse (Association for Computational Linguistics), 27–36.

[B146] Özgür A. Vu T. Erkan G. Radev D. R. (2008). Identifying gene-disease associations using centrality on a literature mined gene-interaction network. Bioinformatics 24, i277–i285. 10.1093/bioinformatics/btn182 18586725 PMC2718658

[B147] Palmer M. Bonial C. Hwang J. D. (2017). VerbNET: capturing English verb behavior, meaning and usage, in The Oxford Handbook of Cognitive Science, 315–336. 10.1093/oxfordhb/9780199842193.013.15. Available online at: https://papers.nips.cc/paper/5021-distributed-representations-of-words-and-phrases-and-their-compositionality

[B148] Panyam N. C. Verspoor K. Cohn T. Ramamohanarao K. (2018a). Exploiting graph kernels for high performance biomedical relation extraction. J. Biomed. Seman. 9, 1–11. 10.1186/s13326-017-0168-3 29382397 PMC5791373

[B149] Panyam N. C. Verspoor K. Cohn T. Ramamohanarao K. (2018b). Exploiting graph kernels for high performance biomedical relation extraction. J. Biomed. Seman. 9:7. 29382397 10.1186/s13326-017-0168-3 PMC5791373

[B150] Peixoto T. P. (2014). The Graph-Tool Python Library. Figshare.

[B151] Peng Y. Gupta S. Wu C. Vijay-Shanker K. (2015). An extended dependency graph for relation extraction in biomedical texts, in Proceedings of BioNLP 15, 21–30.

[B152] Peng Y. Lu Z. (2017). Deep learning for extracting protein-protein interactions from biomedical literature. arXiv [Preprint]. arXiv:1706.01556. 10.18653/v1/W17-2304

[B153] Peng Y. Yan S. Lu Z. (2019). Transfer learning in biomedical natural language processing: an evaluation of BERT and ELMO on ten benchmarking datasets. arXiv [Preprint]. arXiv:1906.05474. 10.18653/v1/W19-5006

[B154] Pennington J. Socher R. Manning C. (2014). Glove: global vectors for word representation, in Proceedings of the 2014 Conference on Empirical Methods in Natural Language Processing (EMNLP), 1532–1543. 10.3115/v1/D14-1162

[B155] Percha B. Altman R. B. (2015). Learning the structure of biomedical relationships from unstructured text. PLoS Comput. Biol. 11:e1004216. 10.1371/journal.pcbi.1004216 26219079 PMC4517797

[B156] Percha B. Altman R. B. (2018). A global network of biomedical relationships derived from text. Bioinformatics 34, 2614–2624. 10.1093/bioinformatics/bty114 29490008 PMC6061699

[B157] Percha B. Garten Y. Altman R. B. (2012). Discovery and explanation of drug-drug interactions via text mining, in Biocomputing 2012 (World Scientific), 410–421. 10.1142/9789814366496_0040 PMC3345566 22174296

[B158] Pesaranghader A. Matwin S. Sokolova M. Pesaranghader A. (2019). deepBIOWSD: effective deep neural word sense disambiguation of biomedical text data. J. Am. Med. Inform. Assoc. 26, 438–446. 10.1093/jamia/ocy189 30811548 PMC7787358

[B159] Peters M. E. Neumann M. Iyyer M. Gardner M. Clark C. Lee K. (2018). Deep contextualized word representations. arXiv [Preprint]. arXiv:1802.05365. 10.18653/v1/N18-1202

[B160] Pletscher-Frankild S. Pallejá A. Tsafou K. Binder J. X. Jensen L. J. (2015). Diseases: text mining and data integration of disease-gene associations. Methods 74, 83–89. 10.1016/j.ymeth.2014.11.020 25484339

[B161] Pylieva H. Chernodub A. Grabar N. Hamon T. (2018). Improving automatic categorization of technical vs. laymen medical words using fasttext word embeddings. Available online at: https://halshs.archives-ouvertes.fr/halshs-01968357/

[B162] Pyysalo S. Ginter F. Heimonen J. Björne J. Boberg J. Järvinen J.. (2007). Bioinfer: a corpus for information extraction in the biomedical domain. BMC Bioinformatics 8:50. 10.1186/1471-2105-8-50 17291334 PMC1808065

[B163] Quan C. Hua L. Sun X. Bai W. (2016). Multichannel convolutional neural network for biological relation extraction. BioMed Res. Int. 2016. 10.1155/2016/1850404 28053977 PMC5174749

[B164] Quan C. Ren F. (2014). Gene-disease association extraction by text mining and network analysis, in Proceedings of the 5th International Workshop on Health Text Mining and Information Analysis, 54–63. 10.3115/v1/W14-1108

[B165] Radford A. Wu J. Child R. Luan D. Amodei D. Sutskever I. (2019). Language models are unsupervised multitask learners. OpenAI Blog 1:9 Available online at: https://arxiv.org/abs/1908.05691

[B166] Ravikumar K. Liu H. Cohn J. D. Wall M. E. Verspoor K. (2012). Literature mining of protein-residue associations with graph rules learned through distant supervision. J. Biomed. Seman. 3:S2. 10.1186/2041-1480-3-S3-S2 23046792 PMC3465209

[B167] Rebholz-Schuhmann D. (2013). Biomedical named entity recognition, whatizit, in Encyclopedia of Systems Biology, 132–134. 10.1007/978-1-4419-9863-7_15118426548

[B168] Rocktäschel T. Weidlich M. Leser U. (2012). Chemspot: a hybrid system for chemical named entity recognition. Bioinformatics 28, 1633–1640. 10.1093/bioinformatics/bts183 22500000

[B169] Rong X. (2014). word2vec parameter learning explained. arXiv [Preprint]. arXiv:1411.2738.

[B170] Routes J. M. Cook J. L. (1995). E1A gene expression induces susceptibility to killing by NK cells following immortalization but not adenovirus infection of human cells. Virology 210, 421–428. 10.1006/viro.1995.1358 7618277

[B171] Sabbir A. Jimeno-Yepes A. Kavuluru R. (2017). Knowledge-based biomedical word sense disambiguation with neural concept embeddings, in 2017 IEEE 17th International Conference on Bioinformatics and Bioengineering (BIBE) (IEEE), 163–170. 10.1109/BIBE.2017.00-61 PMC5792196 29399672

[B172] Sahlgren M. (2006). The Word-Space Model: using distributional analysis to represent syntagmatic and paradigmatic relations between words in high-dimensional vector spaces (Ph.D. thesis). Available online at: http://eprints.sics.se/437/1/TheWordSpaceModel.pdf

[B173] Sahu S. K. Anand A. (2018). Drug-drug interaction extraction from biomedical texts using long short-term memory network. J. Biomed. Inform. 86, 15–24. 10.1016/j.jbi.2018.08.005 30142385

[B174] Sahu S. K. Christopoulou F. Miwa M. Ananiadou S. (2019). Inter-sentence relation extraction with document-level graph convolutional neural network. arXiv [Preprint]. arXiv:1906.04684. 10.18653/v1/P19-1423

[B175] Sarangdhar M. Gudivada R. C. Shrestha R. B. Wang Y. Jegga A. G. (2016). Network analyses of biomedical and genomic big data, in Big Data of Complex Networks (Chapman and Hall/CRC), 13–36. Available online at: https://link.springer.com/chapter/10.1007/978-3-642-22913-8_10

[B176] Schmitz M. Bart R. Soderland S. Etzioni O. (2012). Open language learning for information extraction, in Proceedings of the 2012 Joint Conference on Empirical Methods in Natural Language Processing and Computational Natural Language Learning (Association for Computational Linguistics), 523–534.

[B177] Schwartz A. S. Hearst M. A. (2002). A simple algorithm for identifying abbreviation definitions in biomedical text, in Biocomputing 2003 (World Scientific), 451–462. 10.1142/9789812776303_0042 12603049

[B178] Settles B. (2004). Biomedical named entity recognition using conditional random fields and rich feature sets, in Proceedings of the International Joint Workshop on Natural Language Processing in Biomedicine and Its Applications (NLPBA/BioNLP), 107–110. 10.3115/1567594.156761819623491

[B179] Shardlow M. Nguyen N. Owen G. O'Donovan C. Leach A. McNaught J. (2018). A new corpus to support text mining for the curation of metabolites in the Chebi database, in Proceedings of the Eleventh International Conference on Language Resources and Evaluation (LREC-2018).

[B180] Shen D. Zhang J. Zhou G. Su J. Tan C.-L. (2003). Effective adaptation of a hidden Markov model-based named entity recognizer for biomedical domain, in Proceedings of the ACL 2003 Workshop on Natural Language Processing in Biomedicine (Association for Computational Linguistics), 49–56. 10.3115/1118958.1118965

[B181] Skusa A. Rüegg A. Köhler J. (2005). Extraction of biological interaction networks from scientific literature. Brief. Bioinform. 6, 263–276. 10.1093/bib/6.3.263 16212774

[B182] Smolander J. Dehmer M. Emmert-Streib F. (2019). Comparing deep belief networks with support vector machines for classifying gene expression data from complex disorders. FEBS Open Bio 9, 1232–1248. 10.1002/2211-5463.12652 31074948 PMC6609581

[B183] Song M. Kim W. C. Lee D. Heo G. E. Kang K. Y. (2015). PKDE4J: entity and relation extraction for public knowledge discovery. J. Biomed. Informatics 57, 320–332. 10.1016/j.jbi.2015.08.008 26277115

[B184] Song Q. (2018). An overview of reciprocal l 1-regularization for high dimensional regression data. Wiley Interdiscipl. Rev. Comput. Stat. 10:e1416 10.1002/wics.1416

[B185] Soomro P. D. Kumar S. Banbhrani A. A. S. Shaikh A. A. Raj H. (2017). Bio-NER: biomedical named entity recognition using rule-based and statistical learners. Int. J. Adv. Comput. Sci. Appl. 8, 163–170. 10.14569/IJACSA.2017.081220

[B186] Suárez-Paniagua V. Zavala R. M. R. Segura-Bedmar I. Martínez P. (2019). A two-stage deep learning approach for extracting entities and relationships from medical texts. J. Biomed. Inform. 99:103285. 10.1016/j.jbi.2019.103285 31546016

[B187] Sukthanker R. Poria S. Cambria E. Thirunavukarasu R. (2020). Anaphora and coreference resolution: a review. Inform. Fusion 59, 139–162. 10.1016/j.inffus.2020.01.010

[B188] Swaminathan R. Sharma A. Yang H. (2010). Opinion mining for biomedical text data: feature space design and feature selection, in The Nineth International Workshop on Data Mining in Bioinformatics, BIOKDD.

[B189] Szklarczyk D. Franceschini A. Wyder S. Forslund K. Heller D. Huerta-Cepas J.. (2014). String v10: protein-protein interaction networks, integrated over the tree of life. Nucleic Acids Res. 43, D447–D452. 10.1093/nar/gku1003 25352553 PMC4383874

[B190] Tanabe L. Xie N. Thom L. H. Matten W. Wilbur W. J. (2005). Genetag: a tagged corpus for gene/protein named entity recognition. BMC Bioinformatics 6:S3. 10.1186/1471-2105-6-S1-S3 15960837 PMC1869017

[B191] Tang B. Cao H. Wang X. Chen Q. Xu H. (2014). Evaluating word representation features in biomedical named entity recognition tasks. BioMed Res. Int. 2014. 10.1155/2014/240403 24729964 PMC3963372

[B192] Todeschini R. Consonni V. Mannhold R. (2002). Handbook of Molecular Descriptors. Weinheim: Wiley-VCH.

[B193] Toutanova K. Klein D. Manning C. D. Singer Y. (2003). Feature-rich part-of-speech tagging with a cyclic dependency network, in Proceedings of the 2003 Conference of the North American Chapter of the Association for Computational Linguistics on Human Language Technology (Association for computational Linguistics), 173-180. 10.3115/1073445.1073478

[B194] Trieu H.-L. Nguyen N. T. Miwa M. Ananiadou S. (2018). Investigating domain-specific information for neural coreference resolution on biomedical texts, in Proceedings of the BioNLP 2018 Workshop, 183–188. 10.18653/v1/W18-2324

[B195] Tripathi S. Dehmer M. Emmert-Streib F. (2014). NetBioV: an R package for visualizing large network data in biology and medicine. Bioinformatics 30, 2834–2836. 10.1093/bioinformatics/btu384 24928209

[B196] Tsai R. T.-H. Wu S.-H. Chou W.-C. Lin Y.-C. He D. Hsiang J.. (2006). Various criteria in the evaluation of biomedical named entity recognition. BMC Bioinformatics 7:92. 10.1186/1471-2105-7-92 16504116 PMC1402329

[B197] Tsuruoka Y. Tateishi Y. Kim J.-D. Ohta T. McNaught J. Ananiadou S. (2005). Developing a robust part-of-speech tagger for biomedical text, in Panhellenic Conference on Informatics (Springer), 382–392. 10.1007/11573036_36

[B198] Turian J. Ratinov L. Bengio Y. (2010). Word representations: a simple and general method for semi-supervised learning, in Proceedings of the 48th Annual Meeting of the Association for Computational Linguistics (Association for Computational Linguistics), 384–394.

[B199] Uzuner O. Bodnari A. Shen S. Forbush T. Pestian J. South B. R. (2012). Evaluating the state of the art in coreference resolution for electronic medical records. J. Am. Med. Inform. Assoc. 19, 786–791. 10.1136/amiajnl-2011-000784 22366294 PMC3422835

[B200] Van Mulligen E. M. Fourrier-Reglat A. Gurwitz D. Molokhia M. Nieto A. Trifiro G.. (2012). The EU-ADR corpus: annotated drugs, diseases, targets, and their relationships. J. Biomed. Inform. 45, 879–884. euadr. 10.1016/j.jbi.2012.04.004 22554700

[B201] Vilar S. Friedman C. Hripcsak G. (2017). Detection of drug-drug interactions through data mining studies using clinical sources, scientific literature and social media. Brief. Bioinform. 19, 863–877. vilar2017detection. 10.1093/bib/bbx010 28334070 PMC6454455

[B202] Vishwanathan S. V. N. Schraudolph N. N. Kondor R. Borgwardt K. M. (2010). Graph kernels. J. Mach. Learn. Res. 11, 1201–1242. Available online at: https://d4mucfpksywv.cloudfront.net/better-language-models/language_models_are_unsupervised_multitask_learners.pdf

[B203] Wang S. Zhou W. Jiang C. (2020). A survey of word embeddings based on deep learning. Computing 102, 717–740. 10.1007/s00607-019-00768-7

[B204] Wang X. Zhang Y. Ren X. Zhang Y. Zitnik M. Shang J.. (2018a). Cross-type biomedical named entity recognition with deep multi-task learning. Bioinformatics 35, 1745–1752. 10.1093/bioinformatics/bty869 30307536

[B205] Wang Y. Wang J. Lin H. Tang X. Zhang S. Li L. (2018b). Bidirectional long short-term memory with CRF for detecting biomedical event trigger in fasttext semantic space. BMC Bioinform. 19:507. fasttextbio2. 10.1186/s12859-018-2543-1 30577839 PMC6302454

[B206] Wang Y. Wang L. Rastegar-Mojarad M. Moon S. Shen F. Afzal N.. (2018c). Clinical information extraction applications: a literature review. J. Biomed. Inform. 77, 34–49. 10.1016/j.jbi.2017.11.011 29162496 PMC5771858

[B207] Wang Y. Zheng K. Xu H. Mei Q. (2018d). Interactive medical word sense disambiguation through informed learning. J. Am. Med. Inform. Assoc. 25, 800–808. biowsd4. 10.1093/jamia/ocy013 29584896 PMC6658868

[B208] Wang Z. Lachmann A. Keenan A. B. Ma'ayan A. (2018e). L1000FWD: fireworks visualization of drug-induced transcriptomic signatures. Bioinformatics 34, 2150–2152. 10.1093/bioinformatics/bty060 29420694 PMC6454499

[B209] Weber L. Münchmeyer J. Rocktäschel T. Habibi M. Leser U. (2019). Huner: improving biomedical ner with pretraining. *Bioinformatics*. Available online at: https://academic.oup.com/bioinformatics/article-abstract/36/1/295/5523847?redirectedFrom=fulltext 10.1093/bioinformatics/btz528 31243432

[B210] Wei C.-H. Kao H.-Y. Lu Z. (2012). SR4GN: a species recognition software tool for gene normalization. PLoS ONE 7:e38460. 10.1371/journal.pone.0038460 22679507 PMC3367953

[B211] Wei C.-H. Kao H.-Y. Lu Z. (2015). GNormPlus: an integrative approach for tagging genes, gene families, and protein domains. BioMed Res. Int. 2015. 10.1155/2015/918710 26380306 PMC4561873

[B212] Wei Q. Chen T. Xu R. He Y. Gui L. (2016). Disease named entity recognition by combining conditional random fields and bidirectional recurrent neural networks. Database 2016. 10.1093/database/baw140 27777244 PMC5088735

[B213] Wei Q. Ji Z. Li Z. Du J. Wang J. Xu J.. (2019). A study of deep learning approaches for medication and adverse drug event extraction from clinical text. J. Am. Med. Inform. Assoc. 10.1093/jamia/ocz063 31135882 PMC6913210

[B214] Wishart D. (2014). Foodb: The Food Database. foodb version 1.0.

[B215] Wishart D. Arndt D. Pon A. Sajed T. Guo A. C. Djoumbou Y.. (2014). T3DB: the toxic exposome database. Nucleic Acids Res. 43, D928–D934. 10.1093/nar/gku1004 25378312 PMC4383875

[B216] Wishart D. S. Feunang Y. D. Guo A. C. Lo E. J. Marcu A. Grant J. R.. (2017). Drugbank 5.0: a major update to the drugbank database for 2018. Nucleic Acids Res. 46, D1074–D1082. 10.1093/nar/gkx1037 29126136 PMC5753335

[B217] World Health Organization (2018). International Classification of Diseases. Available online at: https://www.who.int/classifications/icd/en/

[B218] Xing W. Qi J. Yuan X. Li L. Zhang X. Fu Y.. (2018). A gene-phenotype relationship extraction pipeline from the biomedical literature using a representation learning approach. Bioinformatics 34, i386–i394. 10.1093/bioinformatics/bty263 29950017 PMC6022650

[B219] Yadav V. Bethard S. (2019). A survey on recent advances in named entity recognition from deep learning models. arXiv [Preprint]. arXiv:1910.11470. Available online at: http://www.jmlr.org/papers/volume11/vishwanathan10a/vishwanathan10a.pdf

[B220] Yang H. Swaminathan R. Sharma A. Ketkar V. Jason D. (2011). Mining biomedical text towards building a quantitative food-disease-gene network, in Learning Structure and Schemas from Documents (Springer), 205–225. 10.1007/978-3-642-22913-8_10

[B221] Yang Z. Dehmer M. Yli-Harja O. Emmert-Streib F. (2019). Combining deep learning with token selection for patient phenotyping from electronic health records: investigating interpretable vocabularies, sample sizes and architectures. Sci. Rep. 10, 1–18. 10.1038/s41598-020-58178-1 PMC6989657 31996705

[B222] Yoon W. So C. H. Lee J. Kang J. (2019). CollaboNet: collaboration of deep neural networks for biomedical named entity recognition. BMC Bioinform. 20:249. 10.1186/s12859-019-2813-6 31138109 PMC6538547

[B223] Zeng D. Liu K. Lai S. Zhou G. Zhao J.. (2014). Relation classification via convolutional deep neural network. Available online at: https://www.aclweb.org/anthology/C14-1220.pdf

[B224] Zhang C. Biś D. Liu X. He Z. (2019a). Biomedical word sense disambiguation with bidirectional long short-term memory and attention-based neural networks. BMC Bioinform. 20:502. 10.1186/s12859-019-3079-8 31787096 PMC6886160

[B225] Zhang S. Elhadad N. (2013). Unsupervised biomedical named entity recognition: experiments with clinical and biological texts. J. Biomed. Inform. 46, 1088–1098. 10.1016/j.jbi.2013.08.004 23954592 PMC3865922

[B226] Zhang Y. Lin H. Yang Z. Wang J. Sun Y. Xu B.. (2019b). Neural network-based approaches for biomedical relation classification: a review. J. Biomed. Inform. 10.1016/j.jbi.2019.103294 31557530

[B227] Zhang Y. Lin H. Yang Z. Wang J. Zhang S. Sun Y.. (2018a). A hybrid model based on neural networks for biomedical relation extraction. J. Biomed. Inform. 81, 83–92. 10.1016/j.jbi.2018.03.011 29601989

[B228] Zhang Y. Qi P. Manning C. D. (2018b). Graph convolution over pruned dependency trees improves relation extraction. arXiv [Preprint]. arXiv:1809.10185. 10.18653/v1/D18-1244

[B229] Zhang Y. Zheng W. Lin H. Wang J. Yang Z. Dumontier M. (2018c). Drug-drug interaction extraction via hierarchical RNNs on sequence and shortest dependency paths. Bioinformatics 34, 828–835. 10.1093/bioinformatics/btx659 29077847 PMC6030919

[B230] Zhao D. Wang J. Lin H. Yang Z. Zhang Y. (2019). Extracting drug-drug interactions with hybrid bidirectional gated recurrent unit and graph convolutional network. J. Biomed. Inform. 99:103295. 10.1016/j.jbi.2019.103295 31568842

[B231] Zhao Z. Yang Z. Luo L. Lin H. Wang J. (2016). Drug drug interaction extraction from biomedical literature using syntax convolutional neural network. Bioinformatics 32, 3444–3453. 10.1093/bioinformatics/btw486 27466626 PMC5181565

[B232] Zheng J. Chapman W. W. Crowley R. S. Savova G. K. (2011a). Coreference resolution: a review of general methodologies and applications in the clinical domain. J. Biomed. Inform. 44, 1113–1122. 10.1016/j.jbi.2011.08.006 21856441 PMC3226856

[B233] Zheng J. Chapman W. W. Crowley R. S. Savova G. K. (2011b). Coreference resolution: a review of general methodologies and applications in the clinical domain. J. Biomed. Inform. 44, 1113–1122. 21856441 10.1016/j.jbi.2011.08.006 PMC3226856

[B234] Zheng J. Chapman W. W. Miller T. A. Lin C. Crowley R. S. Savova G. K. (2012). A system for coreference resolution for the clinical narrative. J. Am. Med. Inform. Assoc. 19, 660–667. 10.1136/amiajnl-2011-000599 22298565 PMC3384116

[B235] Zheng W. Lin H. Li Z. Liu X. Li Z. Xu B.. (2018). An effective neural model extracting document level chemical-induced disease relations from biomedical literature. J. Biomed. Inform. 83, 1–9. 10.1016/j.jbi.2018.05.001 29746916

[B236] Zhou J. Fu B.-Q. (2018). The research on gene-disease association based on text-mining of pubmed. BMC Bioinformatics 19:37. 10.1186/s12859-018-2048-y 29415654 PMC5804013

[B237] Zhu F. Patumcharoenpol P. Zhang C. Yang Y. Chan J. Meechai A.. (2013). Biomedical text mining and its applications in cancer research. J. Biomed. Inform. 46, 200–211. 10.1016/j.jbi.2012.10.007 23159498

[B238] Zhu Q. Li X. Conesa A. Pereira C. (2017). Gram-CNN: a deep learning approach with local context for named entity recognition in biomedical text. Bioinformatics 34, 1547–1554. 10.1093/bioinformatics/btx815 29272325 PMC5925775

